# Plant Part-Derived Carbon Dots for Biosensing

**DOI:** 10.3390/bios10060068

**Published:** 2020-06-17

**Authors:** Muhammad Zulfajri, Hani Nasser Abdelhamid, Sri Sudewi, Sandhiya Dayalan, Akhtar Rasool, Ahsan Habib, Genin Gary Huang

**Affiliations:** 1 Department of Medicinal and Applied Chemistry, Kaohsiung Medical University, Kaohsiung 80708, Taiwan; u108850005@kmu.edu.tw (S.S.); u107850013@kmu.edu.tw (S.D.); 2 Department of Chemistry Education, Universitas Serambi Mekkah, Banda Aceh 23245, Indonesia; 3 Advanced Multifunctional Materials Laboratory, Department of Chemistry, Faculty of Science, Assiut University, Assiut 71516, Egypt; hany.abdelhamid@aun.edu.eg; 4 Department of Pharmacy, Universitas Sam Ratulangi, Manado 95115, Indonesia; 5 Department of Environmental Sciences, Osmania University, Hyderabad 500007, Telangana, India; akhtarrasool01@gmail.com; 6 Department of Chemistry, University of Dhaka, Dhaka 1000, Bangladesh; habibchem@du.ac.bd; 7 Department of Medical Research, Kaohsiung Medical University Hospital, Kaohsiung 80708, Taiwan; 8 Department of Chemistry, National Sun Yat-sen University, Kaohsiung 80724, Taiwan

**Keywords:** carbon dots, plant parts, natural resources, optical properties, biosensing, biomolecules, molecules, biological samples

## Abstract

Carbon dots (CDs) are a new cluster of carbon atoms with particle size less than 10 nm. CDs also exhibit interesting fluorescence (FL) properties. CDs are attractive because of their fascinating characteristics including low toxicity, good water solubility, and tremendous biocompatibility. Recently, CDs have been investigated as biosensors for numerous target analytes. Meanwhile, the utilization of cheap and renewable natural resources not only fulfills the pressing requirement for the large-scale synthesis of CDs but also encourages the establishment of sustainable applications. The preparation of CDs using natural resources, i.e., plants, offers several advantages as it is inexpensive, eco-friendly, and highly available in the surroundings. Plant parts are readily available natural resources as the starting materials to produce CDs with different characteristics and attractive applications. Several review articles are now available covering the synthesis, properties, and applications of CDs. However, there is no specific and focused review literature discussing plant part-derived CDs for biosensing applications. To handle this gap, we provide a review of the progress of CDs derived from various plant parts with their synthesis methods, optical properties, and biosensing applications in the last five years. We highlight the synthesis methods and then give an overview of their optical properties and applications as biosensors for various biomolecules and molecules in biological samples. Finally, we discuss some future perspectives for plant part-derived CDs for better material development and applications.

## 1. Introduction

Carbon dots (CDs) were initially serendipitously discovered in 2004 as fluorescent (FL) impurities during the purifying of single-walled carbon nanotubes [1]. In 2006, the synthesis of FL CDs using laser ablation and surface passivation was reported [2]. CDs are known as discrete, quasi-spherical particles with a particle size below 10 nm and outstanding FL properties [3]. Research on the fascinating nature of CDs has progressed rapidly. CDs have several advantages including good photostability [4], good solvent dispersion [5], easy functionalization or modification [6], a simple synthesis process [7], tunable FL excitation and emission [8], and high sensitivity and selectivity [9], compared to FL organic dyes and other quantum dots [10,11,12,13,14]. In addition, CDs exhibit robust chemical stability [15], inertness [16], stable colloidal solution [17], and photobleaching resistance [18]. Up to now, the exploration of CDs with extraordinary potential has led to intensive studies. Their excellent biological properties including good biocompatibility and low toxicity [19] provide potential applications in biosensing [20], bioimaging [21], and drug delivery [22].

The synthesis of CDs using green chemistry principles offers several advantages such as low chemical exposure, low cost, renewability of the source, abundant source availability, waste reduction, and the potential to scale up [23]. These green CDs are prepared using green precursors as carbon sources. Plant parts including root, stem, leaf, fruit, flower, and seed have been used in the synthesis of green CDs. Plant parts as natural resources are environmentally friendly materials compared to other resources and have several benefits: they are cheap, easy to obtain, safe, high in abundance, and renewable, providing sustainability [24]. Additionally, the synthesis of CDs from plant parts can transform several low-value materials into functional materials with high biocompatibility [25]. Plant parts containing various heteroatoms such as nitrogen (N) and sulfur (S) are the most appropriate raw starting materials for CDs compared to other carbon sources that require an additional heteroatom sources [26]. The use of plant parts as green resources not only meets the urgent need for large-scale production of CDs, but also promotes the development of sustainable applications. Plant parts do not require a separate reactant for doping, surface passivation, or postmodification due to the existence of numerous carbohydrates, proteins, amino acids, and other biomolecules which provide sufficient elements for the surface functionality of CDs compared to chemical substances as carbon sources [27]. Meanwhile, green synthesis methods are very suitable compared to chemical and physical processes [28]. Various synthesis methods have been performed such as microwave treatment, pyrolysis, hydrothermal treatment, laser ablation, and chemical oxidation [29]. Several plant parts have been utilized in the synthesis of CDs including cranberry beans [30], star fruit [31], kiwi, avocado, pear [19], etc. Therefore, scientists are still inspired to construct new designs using such easily available natural precursors.

The number of scientific articles regarding CDs has increased exponentially [29,32,33,34,35,36,37,38,39,40,41,42,43,44,45,46,47]. However, a focused review of CDs derived only from various plant parts for the specific application as biosensors has still not been made available until now, and this review article aims to fill the vacancy. In this review, we first discuss the progress of plant parts used for the synthesis of CDs with various methods and their optical properties in the past five years. Then, we overview their biosensing applications in the detection of biomolecules and important molecules in biological samples, excluding bioimaging applications. Finally, we provide some future perspectives after observing some of the limitations of CDs from plant parts to enhance their development and potential applications in the future.

## 2. Synthesis Methods for CDs Using Plant Parts

**Figure 1:**
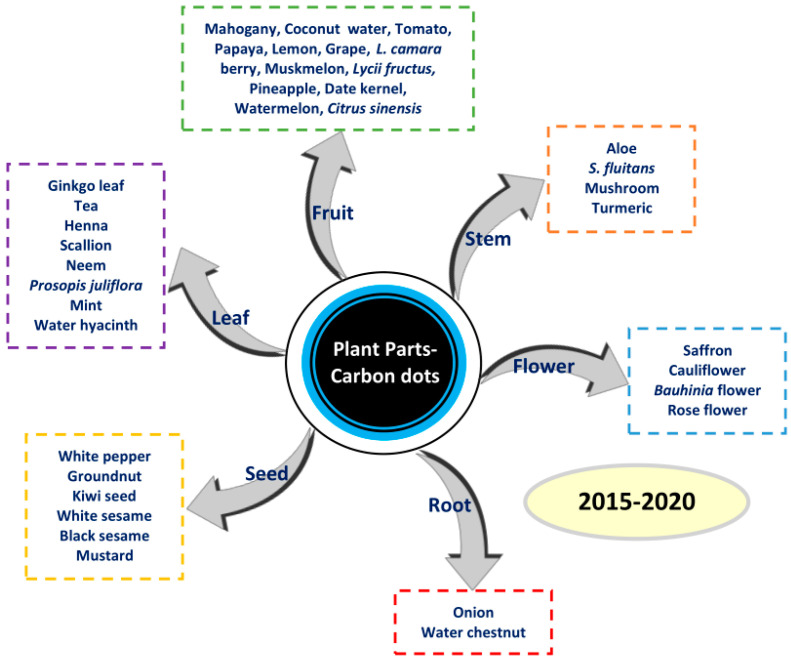
Examples of various plant parts for the synthesis of carbon dots (CDs).

Various synthesis methods have been used and developed since the discovery of CDs in 2004. These synthesis methods can be performed via top-down and bottom-up routes. The top-down method points to the decomposition of larger carbon structures by chemical or physical methods, whereas the bottom-up method points to the transformation of smaller carbon structures into CD nanoparticles through a chemical reaction. Several methods such as chemical oxidation, pyrolysis, and hydrothermal, solvothermal, microwave, and microwave-assisted methods have been used to prepare plant part-derived CDs. Several plant parts from fruits, stems, flowers, roots, seeds, and leaves utilized as starting materials of CDs are depicted in Figure 1. Based on our observation of the literature, the hydrothermal method has been widely used to synthesize plant part-derived CDs. Here, we discuss the above-mentioned synthesis methods for obtaining plant part-derived CDs. The features of the various synthesis methods used for the synthesis of plant part-derived CDs are resumed in Table 1.

### 2.1. Hydrothermal Method

**Figure 2:**
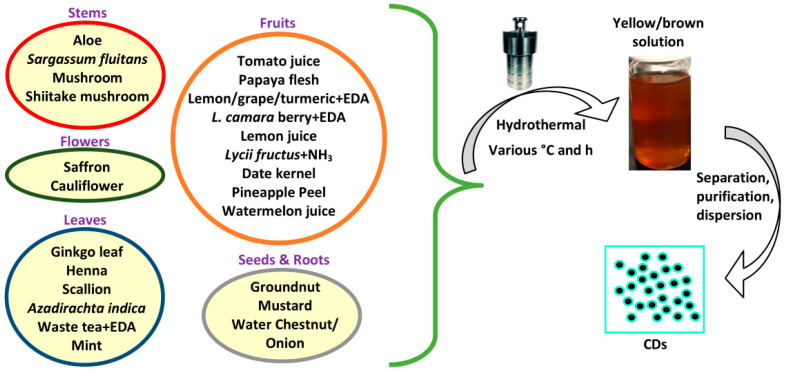
A synthetic procedure scheme for plant part-derived CDs by the hydrothermal treatment method.

The hydrothermal method is a cheap, eco-friendly, and safe method for synthesizing CDs from natural resources. In a typical procedure, a mixture of precursors is placed in a Teflon-lined autoclave and the temperature is kept at between 150 and 250 °C for several hours. The solution color changes to a brown or yellow color, indicating the formation of CDs. Then, the CD solution is separated and purified. An oven or furnace is used for heating purposes. The synthesis process can avoid multistep passivations and expensive reagents, required for sophisticated instruments [38]. Figure 2 depicts an illustration of a hydrothermal method for preparing CDs from several plant parts. Several researchers have utilized the hydrothermal method to prepare CDs from several plant parts. From stem parts, Xu et al. synthesized CDs by using aloe [48]. A total of 5.0 g aloe was dispersed in 25 mL water before heating at 180 °C for 11 h as the optimum hydrothermal conditions. Godavarthi and coworkers synthesized nitrogen (N)-CDs from waste seaweed *Sargassum fluitans* [49]. They put 25 g of seaweed in an Erlenmeyer flask, stirred with 2.0 L of distilled water, and repeated eight times after every 30 min, stirring to remove salts and other soluble impurities, followed by drying and pulverizing. Then, 10 g of seaweed powder was dispersed in 100 mL of water and refluxed for 30 min followed by filtration, and 10 mL of seaweed extract mixed with 50 mL water was heated at 180 °C for 5 h. Yang et al. performed the synthesis of CDs from fungus (mushroom) [50]. After drying at 80 °C for 24 h, the mushrooms were crushed into powder. A mixture containing 0.6 g powder and 6 mL ultrapure water was placed into an autoclave and kept at 200 °C for 6 h. Moreover, Wang et al. prepared CDs using dehydrated shiitake mushroom [51]. The shiitake mushroom was soaked and washed by distilled water to diminish the impurities. After freeze drying in a vacuum, the dried shiitake mushroom was ground into powder. A total of 0.5 g powder and 10 mL water were placed into an autoclave. The mixture was reacted at 200 °C for 12 h. From the flowers, Ensafi and coworkers used dried stigma saffron to produce CDs [52]. Saffron was crushed into powder. The mixture of 0.5 g saffron powder with 100 mL doubly distilled water (DDW) was transferred into an autoclave and hydrothermally reacted at 200 °C for 14 h. Tafreshi et al. prepared CDs using cauliflower juice [53]. The cauliflower plant was washed with distilled water and then extracted. The mixture was filtered to eliminate large aggregates, and 60 mL of extract was reacted at 120 °C for 5 h. Using seeds, Roshni synthesized both CDs and N-CDs from groundnuts [54]. An amount of 1 g of groundnut was rinsed, dried, crushed, and dispersed in 30 mL distilled water. The mixture was heated at 250 °C for 6 h. Ethylenediamine (EDA) was utilized as an N dopant to produce N-CDs. Candra et al. synthesized CDs from mustard seeds [55]. First, 2.0 g of mustard seeds were ground and dispersed in 50 mL ultrapure water. The mixture was then poured into an autoclave and reacted at 180 °C for 4 h. Hu et al. produced S- and N-CDs derived from water chestnut powder (nitrogenous compound) and onion powder (thiol compound) as dual precursors. Fresh water chestnut and onion were crushed into a powder, and then 2.0 g of water chestnut powder and 3.0 g of onion powder were diluted in 30 mL ultrapure water and heated at 180 °C and 4 h [56]. For all the above procedures, separation and purification processes using centrifugation, a membrane filter, and dialysis were carried out to obtain pure CDs. All the above reported works were concerned with the synthesis of CDs from plant parts of stems, flowers, seeds, and roots.

Furthermore, the synthesis of CDs from various fruits has been carried out. Miao et al. reported the synthesis of CDs from tomato juice (10 mL) at 150 °C and 2 h reaction time [57]. Wang et al. prepared ethanol-soluble CDs (E-CDs) and water-soluble CDs (W-CDs) with papaya flesh powder (0.4 g) [58]. Papaya peel was discarded and the flesh was chopped into small sections followed by drying at −54 °C for 24 h. They used 10 mL of 90% ethanol and deionized (DI) water as solvents. The mixtures were reacted at 200 °C for 5 h. Hoan et al. prepared CDs from lemon juice (15 mL of extract and 10 mL of water) with the hydrothermal conditions of 240 °C temperature and 12 h reaction time [59]. Ahmadian et al. synthesized N-CDs from grape, lemon, and turmeric extract [60]. A total of 1.0 g extract, 40 mL DI water, and EDA were mixed, stirred for 30 min, and reacted at 180 °C for 6 h. Further, *Lantana camara* berries and EDA were employed as C and N sources for N-CDs by Bandi et al. [61]. First, 5.0 g blue black berries were juiced with 50 mL of DDW water. Then, 20 mL fruit juice was mixed with EDA and reacted at 180 °C for 3 h. Sun et al. prepared N-CDs from *Lycii fructus* [62]. First, 1.0 g *Lycii fructus* was crushed, dispersed in 30 mL ultrapure water, and filtered through a vacuum to obtain a purple product, and then 1 mL of 25% NH_3_ solution was mixed into the mixture and heated at 200 °C for 5 h. Amin et al. prepared CDs from date kernel [63]. The date kernels were rinsed to remove dust and dried under sunlight. Then, they were crushed into a fine powder; 2.0 g date kernel powder was dispersed in 10 mL DI water, stirred for 30 min, and heated at 200 °C for 8 h. Vandarkuzhali et al. prepared CDs using pineapple peel [64]. After rinsing several times, the pineapple peel was made into juice. Then, 10 mL ethanol was added into 10 mL peel extract and placed into an autoclave for heating at 150 °C for 2 h. Lastly, Lu et al. hydrothermally synthesized N-CDs from watermelon juice [65]. They combined 50 mL watermelon juice extract and 5 mL ethanol in an autoclave and heated at 180 °C for 3 h. Filtration and purification were also carried out in all the above cases to obtain purified CDs.

Several leaves have also been used to synthesize CDs. Jiang et al. used ginkgo leaf to prepare N-CDs via a hydrothermal method [66]. In this process, 1.0 g leaf powder was dispersed in 25 mL water and placed into the autoclave for reaction at 200 °C for 10 h. Moreover, Shahshahanipour et al. used *Lawsonia inermis* (henna) leaf powder to prepare CDs [67]. An amount of 0.5 g henna leaf powder was mixed with 40 mL DI water and heated at 180 °C for 12 h. Zhang et al. prepared N- and S-CDs using scallion leaves [68]. A total of 4.0 g leaf powder and 20 mL water were mixed well and placed into the autoclave to heat at 180 °C for 12 h. Yadav et al. prepared CDs from *Azadirachta indica* (neem) leaf [69]. Here, 10 g neem leaf powder was dispersed in 100 mL DI water and heated at 150 °C for 4 h. Chen and coworkers reported the synthesis of CDs from waste tea extract [70]. In their study, 1.5 g waste tea, immersed in 30 mL of DI water and mixed with EDA, was reacted at 150 °C for 6 h to produce two tea CDs. Compared to waste tea extract alone, waste tea extract plus EDA exhibited CDs with higher FL properties. Finally, Raveendran et al. [71] obtained CDs from fresh mint leaves. A total of 5.0 g fresh mint leaves were used to extract the water soluble portion with 40 mL DI water and heated at 200 °C for 5 h. Separation and purification processes were performed for all the above procedures to obtain the purified CDs. The hydrothermal conditions of all the above-mentioned plant part-derived CDs including their precursors, parts, forms, precursor amounts, solvent volumes, hydrothermal temperatures, and reaction times are summarized in Table 2. Such methods for the extensive use of plant parts prevent the production of waste materials and make better use of meaningful sources. Even though hydrothermal treatment to synthesize CDs is low-cost, eco-friendly, non-toxic, and wasy to use, it has disadvantages including poor size control and long reaction times [72,73]. Therefore, increasing the products and adjusting the size of the particles still offer substantial opportunities for exploration elsewhere.

### 2.2. Solvothermal Method

**Figure 3:**
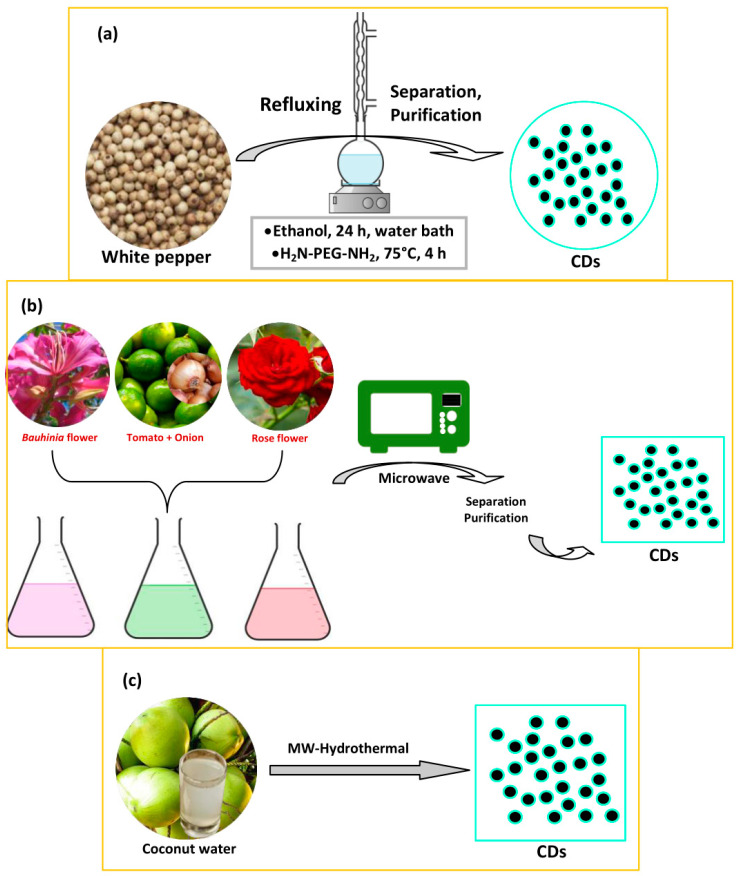
Synthetic procedure illustration of plant part-derived CDs by (**a**) solvothermal, (**b**) microwave, and (**c**) microwave-assisted hydrothermal methods.

The solvothermal method is a cheap, environmentally friendly, and non-toxic method for synthesizing CDs, as with the hydrothermal method. Ideally, a solution of carbon precursors is reacted and sealed in a reactor using high temperatures. The precursors are heated in an organic solvent with a high boiling point, followed by the extraction and concentration procedures [32]. The convenient synthesis process and controllable heteroatom doping make this method an fruitful approach for producing CDs. There are several drawbacks from this method, namely carbonaceous aggregation during carbonization, the difficulty of size control, and the solubility problem [32]. There was a report of the use of the solvothermal method for creating plant part-derived CDs to act as a biosensor. Long and coworkers synthesized dual-emission CDs from white pepper through the one-pot solvothermal method [74]. White pepper was refluxed in anhydrous ethanol under N_2_ flow. Typically, white pepper was dried at 60 °C and ground into powder. Then, 2.0 g white pepper powder was dispersed in 40 mL anhydrous ethanol in a round-bottom flask followed by refluxing in a water bath for 24 h under N_2_ flow. After filtration, H_2_N-PEG-NH_2_/anhydrous ethanol solution (1.2 mg mL^−1^/10 mL) was mixed into the filtrate and continually refluxed at 75 °C for 4 h. The resulting CDs were further filtered and freeze-dried to obtain powder CDs. An illustration of the solvothermal method for preparing CDs can be seen in Figure 3a.

### 2.3. Microwave Treatment

Microwave treatment is a streamlined, scalable, low-cost, eco-friendly, and rapid way to produce CDs [72,75]. Microwave radiation offers fast in situ temporary heating which can dramatically correct both product yields and the quality of the CDs [76]. To date, microwave radiation treatment has been frequently used, but less than the hydrothermal method. Typically, the plant parts are dispersed in a solvent and the mixture is afterward reacted in a microwave oven as shown in Figure 3b. The resulting CDs are then separated and purified. Practically, Huang et al. adopted the *Bauhinia* flower to prepare N-CDs through the microwave method [77]. An amount of 10 g dried *Bauhinia* flower mixed with 50 mL ethanol:water (1:1, *v*/*v*) was transferred into the microwave equipment with irradiation at 1000 W power for 10 min. Monte-Filho et al. synthesized CDs from lemon and onion biomasses in a domestic microwave [78]. The onion was used as an S-dopant, while NH_4_OH was used as an N-dopant. The lemon juice (20 mL) and onion juice (20 mL) were dissolved in 8.0 mL DI water and 10 mL of 25% NH_4_OH solution. The mixture was transferred to an Erlenmeyer flask to irradiate at 1450 W for 6 min. Then, the brownish-black solid was dissolved in DI water, followed by separation and purification. Finally, Feng et al. prepared CDs from rose flower by microwave treatment [79]. Typically, rose flowers were dried at 60 °C and ground into powder. The mixture of 10 mg rose flower petal powder, 2.5 g P_2_O_5_, and 10 mL ultrapure water was stirred together for 10 min. P_2_O_5_ was used as a phosphor (P) dopant. After that, the solution was placed into the microwave oven and heated until the color changed from red to brown. Filtration and purification were performed for gaining the final product. Although the microwave treatment has several useful advantages, its poor control of size is still a drawback of this method [80].

### 2.4. Microwave-Assisted Hydrothermal Method

A microwave treatment is used to heat water in the process of the microwave-assisted hydrothermal method, which takes little time [25]. However, the microwave-assisted hydrothermal method is infrequently used compared to the hydrothermal method. An illustration of this method is depicted in Figure 3c. For practical synthesis, Rahul and Paria used a microwave-assisted hydrothermal method to produce CDs from tender coconut water without adding any other chemicals [81]. This hydrothermal treatment was carried out using a microwave reactor irradiated at 800 W. Every reaction was performed in a sealed glass vessel. The proper vessel temperature was controlled by an Aluminum Trailer Company (ATC) fiber-optic sensor. A mixture of 10 mL of coconut water and ethanol (1:1, *v*/*v*) was transferred into the vessel and placed into the microwave oven to irradiate at various temperatures for 1 min. Finally, the CD solution was purified.

### 2.5. Pyrolysis Treatment

**Figure 4:**
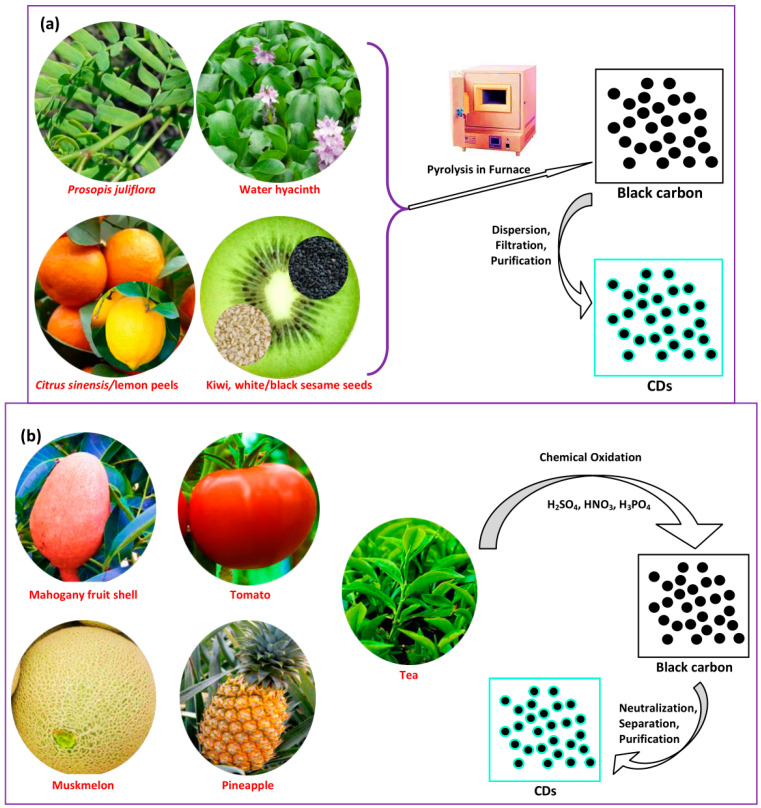
Illustration of the synthesis of plant part-derived CDs by (**a**) pyrolysis and (**b**) chemical oxidation methods.

Pyrolysis treatment is a well-known method for producing CDs. It is an irreversible thermal decomposition reaction in which the plant part materials decompose in an inert atmosphere. It involves physical and chemical changes of materials, producing solid black carbon residue [82]. This method has the benefits of being rapid, simple, and eco-friendly, but is difficult to scale up and produces a broad size distribution [73]. Commonly, pyrolysis is performed at very high temperatures and under controlled pressure, forming black carbon materials that can be separated and purified to obtain the CDs. Pourreza and Ghomi synthesized CDs from *Prosopis juliflora* leaves via a pyrolysis treatment [83]. An amount of 1.0 g *Prosopis juliflora* leaves were washed with water, placed into a crucible, and heated at 200 °C for 30 min followed by crushing into powder and heating at 200 °C for 1 h. Each 0.1 g of the black carbonized powder was dispersed in 10 mL of 50% acetic acid and in ethanol and allowed to sit for 24 h. Subsequently, separation and drying processes were carried out to obtain the CDs. Li et al. prepared N-CDs utilizing kiwi, white sesame, and black sesame seeds [84]. A total of 15 g seeds were transferred into a crucible, individually marked, and heated at 350 °C for 10 h in a furnace, producing the black material products. The products were crushed to powder and washed in dichloromethane (DCM) to eliminate impurities. An N_2_ stream was used to evaporate the DCM. The black solute became yellowish-brown after evaporating and then vanished immediately. Finally, black residue was received and subjected to the separation and purification. Chatzimitakos et al. used *Citrus sinensis* and *Citrus limon* peel to produce two types of CD [85]. The fresh peel was rinsed with DDW water and cut into small sections, put in different porcelain crucibles and heated at 180 °C for 2 h. The homogeneous black residue was crushed in a mortar into a fine powder. The powders of each citrus were mixed with DDW water and sonicated for 10 min. Then, the dispersion was separated and purified followed by freeze-drying. Moreover, Prathumsuwan et al. utilized acid-assisted pyrolysis to synthesize CDs from water hyacinth leaves [86]. The water hyacinth leaves were rinsed with water and ethanol, sun-dried, and crushed into a fine powder. Then, 10 g powder was dispersed in 75 mL of 1.5 M nitric acid (HNO_3_) and 75 mL DI water, followed by refluxing at 110 °C under stirring at 900 rpm for 24 h. The brown solution was heated at 250 °C for 6 h. The resulting black solid was dispersed in DI water and underwent separation and purification to obtain the CDs. A schematic of the synthesis procedure of pyrolysis is depicted in Figure 4a.

### 2.6. Chemical Oxidation

Chemical oxidation is performed to exfoliate and decompose bulk carbon into small particles, and concurrently to incorporate hydrophilic groups such as hydroxyl (-OH) groups or carboxyl (-COOH) groups, which could increase their water dispersion and FL properties [87,88]. The utilization of the chemical oxidation method is convenient and scalable in the production of CDs not requiring complicated equipment [89]. This method is the most common top-down route for the preparation of CDs because of benefits including high yield, high purity, cheapness, and the simple control of size [82]. Chemical oxidants such as HNO_3_, phosphoric acid (H_3_PO_4_), and sulfuric acid (H_2_SO_4_) can be used to facilitate the carbonization of plant parts for CDs. The chemical oxidation procedure for several plant parts for the synthesis of CDs is schematically represented in Figure 4b. Generally, the plant parts are firstly carbonized and the product is subsequently mixed with a chemical oxidant followed by the processes of separation and purification. Gunjal et al. prepared CDs by the oxidation of mahogany fruit shell powder using H_2_SO_4_ and HNO_3_ [90]. The activated carbon was produced from mahogany fruit shell by the proper reaction of dried shell powder and concentrated H_2_SO_4_ followed by neutralization. Then, 0.5 g activated carbon was dispersed in 50 mL 1.0 M HNO_3_ solution and sonicated for 10 min. The mixture was refluxed for 12 h, showing a color change to brownish-yellow, representing the forming of CDs. Sodium carbonate (Na_2_CO_3_) was employed for the neutralization process. The FL could be tuned by surface modification with amines. Gunjal et al. also prepared N-CDs derived from waste tea residue [91]. Waste tea residue powder was dried after being obtained at ambient temperature to maintain other carbonaceous materials such as sugar and milk and crushed into powder. Then, 2.0 g fine powder was placed into a round-bottom flask containing 0.1 M HNO_3_ and refluxed for 6 h. The supernatant was neutralized using Na_2_CO_3_.

Desai and coworkers prepared CDs from muskmelon/*Cucumis melo* (CM) fruit by acid oxidation [92]. The peel was discarded from the CM fruit, which was then cut into small sections. These fruit sections were kept in a freezer at −20 °C. An amount of 10 mL of 34 N H_2_SO_4_ was poured into a beaker containing 0.5 g aqueous freeze-dried CM fruit to produce blue CM CDs. The sonication of the mixture was carried out for 5 min and it was then reacted at 100 °C for 1 h. Subsequently, 2.0 M NaOH was used to neutralize the pH of the CM CD residue. Additionally, 0.5 g freeze-dried CM fruit was dispersed in 5 mL water and 10 mL of 40 N H_3_PO_4_ to produce the green and yellow CM CDs with heating at 80 °C for 25–30 min and 15–20 min, respectively. The neutralization of the final product was performed using 1.0 M NaOH and this was then purified. Kailasa et al. synthesized blue, green, and yellow CDs from tomato by chemical oxidation [93]. H_2_SO_4_ and H_3_PO_4_ were used as chemical oxidants. A total of 100 g tomato was rinsed and then cut into small sections. The tomato sections were stored in a freezer at −20 °C. To synthesize blue CDs, 0.5 g tomato sections were placed into a beaker containing 5 mL DI water, and 10 mL of 34 N H_2_SO_4_ was added slowly into it followed by sonication. The mixture was reacted at 100 °C for 1 h. Then, 10 mL of 40 N H_3_PO_4_ was added and reacted at 80 °C for 25 min to produce green CDs. Yellow CDs were produced by treating the freeze-dried tomato with 10 mL of 40 N H_3_PO_4_ and the mixture was reacted at 80 °C for 20 min. All products were neutralized with NaOH and then purified. Anilbhai et al. also prepared three CDs from pineapple through acid oxidation using H_2_SO_4_ and H_3_PO_4_ [94]. Briefly, the pineapple peel was discarded and sliced into small sections. The pineapple sections were stored at −23 °C. Then, 0.5 g pineapple sections were placed into a flask containing 5.0 mL DI water and sonicated for 5 min. An amount of 10 mL of 34 N H_2_SO_4_ was added to the mixture and reacted at 100 °C for 60 min to produce blue CDs. Then, the mixture was kept aside for a while to achieve room temperature. The reaction mixture became black and was neutralized using 1.0 M NaOH. Green and yellow CDs were prepared in a similar way to blue CDs. Amounts of 10 mL of 40 N H_3_PO_4_/5 mL of water and 10 mL of 40 N H_3_PO_4_ were used for green and yellow CDs, respectively, and were reacted at 80 °C for 20–30 min (green CDs) and 20 min (yellow CDs). Although the surface properties of CDs prepared through chemical oxidation can be easily modified, a major downside of this method is that residue may stay in the CDs, which could elevate biological toxicity, going against the main rationale for utilizing green CDs [76]. It also requires complicated post-treatment and uses expensive oxidants. Its rate of production is mostly intermittent, with a low production capacity [25]. So far, the utilization of chemical oxidation has been scarcely used in producing CDs from plant parts as carbon precursors.

## 3. Optical Properties of Plant Part-Derived CDs

**Figure 5:**
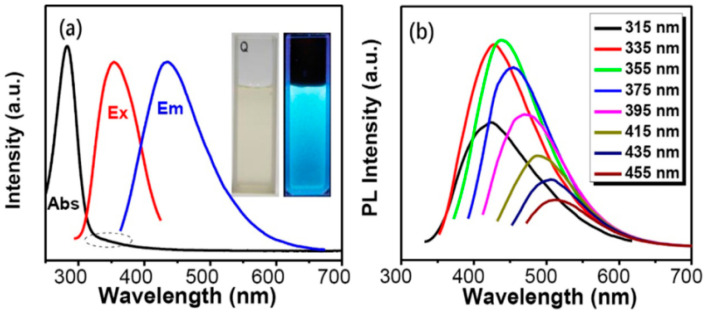
(**a**) UV-vis absorbance (Abs), FL excitation (Ex) (355 nm), and emission (Em) (439 nm) spectra of N-CDs (inset: pictures of CD solution irradiated with daylight and UV light). (**b**) Excitation-dependent emission with the excitation wavelength of 315–455 nm. The figure is reprinted with permission from Ref. [65]. Copyright belongs to Elsevier. Abbreviations: FL: fluorescence.

The main elements of plant part-derived CDs are C, O, H, and N atoms, which present in various functional groups and provide good water solubility. The carbon sources are mainly citric acid (CA), amino acids, ascorbic acid, polysaccharides, carbohydrates, proteins, and other small molecules [95]. When these carbon sources are used for the synthesis of CDs, most of them do not need the addition of a substance for doping, but rather self-dope [25]. Mechanistically, the optical absorbance and FL emission of CDs do not come from a band-gap but are induced by p-plasmon and the radiative recombination of surface-confined electrons and holes, distinct from that of traditional semiconductor quantum dots [13,96,97,98,99]. Quantum effects and defects are presently assumed to be the primary emissive mechanisms of CDs [100]. Exceptional efforts have been dedicated to investigate the origin of FL, although the mechanism is still not clear. Here, we discuss the optical absorption and FL properties of plant part-derived CDs that later were used as biosensors. Figure 5a,b gives an example of the UV-vis absorbance, color, optimum excitation and emission peaks, and spectra of emission-dependent excitation of plant part-derived CDs (from watermelon juice).

### 3.1. UV-vis Absorbance and Emission Color

In general, almost like other CDs, the UV-vis absorbance spectra of plant part-derived CDs usually have a strong peak in the UV area with a tail expanding into the visible area as depicted in Figure 5a. Most plant part-derived CDs exhibit absorbance in the range of 280–360 nm. Most plant part-derived CDs have insignificant absorbance in the visible region, but they can emit a full color from blue to red according to the specific CD. The absorbance at 230–270 nm is correlated to C=C bonds with a π-π* transition and the shoulder peak at around 300–330 nm is correlated to C=O/N=O bonds with an n-π* transition [101]. Several plant part-derived CDs show only one absorption peak between 270 and 302 nm, which is associated with the π-π* transition (C=C bonds) and the n-π* transition (C=O bonds). Plant part-derived CDs from *Citrus sinensis/limon* peels, saffron, date kernel, aloe, groundnut, cauliflower, lemon juice, pineapple peel, scallion, mushroom, coconut water, mahogany fruit shell, waste tea residue, and papaya flesh exhibited an absorption peak at about 270, 275, 275, 278, 279, 280, 280, 280, 281, 285, 290, 300, 302, and 250–290 nm, respectively (Table 3). The CDs derived from date kernel [63], groundnuts [54], and pineapple peel [64] exhibited absorbance peaks at 275, 279, and 280 nm, extending until the near-visible range associated with the aromatic C=C bonds of the π-π* transition. The UV-vis absorbance of CDs from lemon juice showed the absorption peak at 280 nm with a tail expanding to the visible region associated with the conjugated C=O bond transition [59]. CDs derived from scallion leaf showed a distinct peak at 281 nm which derives from C=C or C=N bonds of the π-π* transition [102]. Moreover, the CDs from citrus peels [85], saffron [52], aloe [48], cauliflower [53], and mahogany fruit shell [90] exhibited a sharp absorption peak at 270, 275, 278, 280, and 300 nm, respectively, which can be assigned to C=O bonds with an n-π* transition and C=C bonds with a π-π* transition [103]. The coconut water-derived CDs [81] exhibited a high absorbance peak at 290 nm with a tail expanding to the visible area associated with the existence of aromatic π orbitals due to the forming of graphitic carbon structure [104]. Meanwhile, the UV-vis absorbance of CDs derived from mushroom [50] and waste tea residue [91] exhibited a peak at 285 and 302 nm, respectively, correlating to C=O bonds with an n-π* transition [105]. Papaya flesh CDs showed maximum absorption at wavelengths ranging from 250 to 290 nm, correlating to the existence of an aromatic π orbital or n-π* transition [106].

Furthermore, CDs from *Bauhinia* flower [77], ginkgo leaf [66], *Lycii fructus* [62], and *Sargassum fluitans* [49] showed clearly two absorption peaks below 300 nm at 228/282, 230/280, 271/300, and 226/280 nm, respectively. *Bauhinia* flower- and ginkgo leaf-derived CDs exhibited two peaks related to C=C sp^2^ domains with a π-π* transition and a C=O bond with an n-π* transition [107,108]. The CDs from *Lycii fructus* showed two peaks referring to C=N/C=C bonds with a π-π* transition and C=O or amine groups with an n-π* transition [109,110]. Conversely, CDs from *Sargassum fluitans* displayed their two bands at 226 and 280 nm, from the core section of carbon as an n-π* transition and from the surface/molecular section as a π-π* transition [111]. Next, several plant part-derived CDs exhibited two distinct peaks below and above 300 nm, such as *Lantana camara* berries [61], watermelon juice [65], lemon/onion [78], neem leaf [69], waste tea [70], water hyacinth [86], kiwi/white sesame/black sesame seeds [84], mustard seeds [55], and water chestnut/onion [56], which had absorption peaks at 285/356, 282/355, 280/340, 276/340, 270/330, 285/350, 275/325, 245/312, and 242/333 nm, respectively. The peaks below and above 300 nm were ascribed to π-π* transitions of the aromatic C=C or C=N sp^2^ carbon domains and an n-π* transition of C=O bonds [112,113]. For N- and S-CDs from water chestnut/onion, the peak at 333 nm was based on a trapping excitation strategy [114].

CDs derived from muskmelon fruit [92], mint leaf [71], tomato [93], and pineapple [94] showed three distinct peaks. Three CDs from muskmelon fruit showed peaks at 314, 414, and 467 nm of π-π* and n-π* electronic transitions because of some surface-state functionalities (-NH_2_, -OH, and -COOH) on the CDs. Meanwhile, CDs from mint leaf showed three peaks at 225, 281, and 323 nm where those at 225 and 281 nm can be attributed to C=C bonds with π-π* transitions and that at 323 nm attributed to C=O bonds with n-π* transitions. Three CDs from tomato exhibited the optimum absorbance at 260, 280, and 285 nm, associated with sp^2^ carbons in aromatic compounds with a π-π* transition and C=O/C=N bonds with an n-π* transition, respectively. These absorbance peaks reveal the presence of π-π* and n-π* transitions because of the presence of -CHO, -C=O, -COOH, and NH_2_ functional groups. Also, the absorbance of three CDs derived from pineapple appeared at 318, 395, and 393 nm, related to π-π* and n-π* transitions. Interestingly, CDs from white pepper showed four absorbance peaks: at 261, 310, and 343 nm, and a small one at 665 nm. The absorbance peak at 261 nm can be attributed to the aromatic sp^2^ domains with a π-π* transition. The absorbance peaks at 310 and 343 nm were same as the typical absorbances of piperine contained in white pepper [115], which could indicate that an N element was doped in the CDs. The absorbance band at 665 nm is related to the Q-band of porphyrin derivatives.

Moreover, CDs derived from shiitake mushroom [51], henna leaf [67], and *Prosopis juliflora* leaf [83] showed absorption peaks ranging from 240–290/420–500, 270–380, and 300–500 nm, respectively. The CDs from shiitake mushroom have a small absorbance in the range of 420–500 nm because of the surface states of the CDs, and the absorption at 240–290 nm is correlated to the C=C bond with a π-π* transition and the C=O bond or pyridine nitrogen with an n-π* transition. The CDs from henna leaf had wide absorbance peaks from 270 to 380 nm. These bands can differentiate the existence of the specific orbitals of the π-system and the extended conjugation of C=C and C=O bonds through π-π* and n-π* transitions. The presence of a clear tail in the visible region may be because of the small size of the particles and Mie scattering [116]. In addition, CDs derived from *Prosopis juliflora* leaf showed clearly a broad peak at 300–500 nm related to the π-π* or n-π* transitions. The CDs from lemon/grape/turmeric/EDA showed an absorbance peak at 350 nm that contributes to the π-π* transition. For the color of plant part-derived CDs under UV light, almost all CDs exhibited a (bright) blue color, except for several CDs. Saffron- and water chestnut/onion-derived CDs produced a green-blue color while CDs from coconut water exhibited a blue and green color under UV light irradiation. Three CDs from tomato, muskmelon, and pineapple showed blue, green, and yellow colors. Moreover, the CDs from lemon juice and henna leaf emitted bright green and green colors. In addition, mint leaf CDs showed a cyan color when irradiating with UV light. The green-blue FL emission of the water chestnut- and onion-derived S- and N-CDs originates from S atom doping. Therefore, almost all plant part-derived CDs exhibited two absorption peaks of π-π* and n-π* transitions and a blue emission color under UV light irradiation, proving the FL properties of plant part-derived CDs. The summary of the UV-vis absorbances and emission colors can be seen in Table 3.

Many CDs could emit emission colors from blue to red, but most of them emitted strong blue and green colors, which are not preferred for bio-applications [117]. Ideally, CDs should show continually adaptable excitation-dependent full-color emission, where they can create any visible color by selecting various wavelengths without shifting the chemical structure. The full-color emission of the CDs is really correlated to the C=O and C=N functional groups or others, which can produce multiple structural configurations and present a new level of energy in their electronic structures, generating possibilities for electronic transition [118]. CDs with colorful emissions can originate from diverse sizes, in which the emissions red-shift progressively with longer reaction times and larger size [119]. Currently, there are several reported methods for obtaining multicolor emissions from CDs, such as tuning the reaction time or temperature, introducing conjugated precursors, surface modification, heteroatom doping, using different solvents, and adding acidic chemicals [118,120,121,122,123,124,125]. Alterations in emission color can also be caused by using different isomers such as phenylenediamines [126]. The control of surface states is the most major and convenient method for regulating the colors emitting from CDs. Diverse functional groups can establish numerous surface defect states, presenting substantial energy level band gaps that modulate the emission of color from the CDs. The N-containing functional groups generated by doping will have a significant effect on the colors emitted from CDs [122]. Therefore, the above-mentioned methods can be used to prepare plant part-derived CDs with full-color emission properties.

### 3.2. Fluorescence and Quantum Yield

Plant part-derived CDs exhibited attractive FL properties with excitation-dependent emission behavior, which is almost the same as other CDs. Although several suggestions regarding the FL mechanism of CDs have been proposed, such as emissive traps, quantum-confinement effects, free zig-zag sites, and electronic conjugate structures, the FL mechanism of CDs remains an open question [38]. It will be useful for the selection of appropriate CDs in biosensing through coherent analysis. In most literature, the optimum excitation and emission have been explored as optical properties. Different CDs will exhibit distinct optimum excitation and emission wavelengths. Most of them showed an optimum excitation wavelength between 300 and 400 nm and emission wavelength between 400 an 500 nm with a quantum yield (QY) below 50%.

Several plant part-derived CDs have different highest emission wavelengths under a 350 nm excitation wavelength. The maximum emission wavelength of CDs from waste tea residue at 430 nm appeared by excitation at 310 nm with a QY of 2.47% [91]. The emission spectra closely correlated to the excitation wavelengths of 300–350 nm, with an emission shift, showing excitation-dependent emission behavior. The CDs derived from scallion leaf [68] and mahogany fruit shell [90] showed optimum emission wavelengths at 418 and 430 nm, respectively, with a 320 nm excitation wavelength. The FL emission peak progressively red-shifted when changing the excitation wavelength from 300 to 400 nm and 290 to 350 nm for scallion CDs and mahogany fruit shell CDs with a QY of 3.2% and 1.9%, respectively. Meanwhile, the CDs from cauliflower juice [53], shiitake mushroom [51], and mustard seeds [55] showed excitation at 325, 330, and 330 nm with optimum emission wavelengths at 400, 410, and 423 nm, respectively. The cauliflower juice CDs also exhibited an optimum emission peak at the excitation wavelength of 350 nm. The cauliflower juice and shiitake mushroom CDs emitted FL spectra in the ranges of 300–600 and 410–500 nm, followed by a progressive lowering of their FL intensity. The QYs were 43%, 5.5%, and 4.6% for the CDs from cauliflower juice, shiitake mushroom, and mustard seeds, respectively. In addition, the CDs derived from lemon/onion [78], date kernel [63], *Sargassum fluitans* [49], and neem leaf [69] showed maximum emission wavelengths at 425, 430, 450, and 467 nm, respectively, with a 340 nm excitation wavelength. With the excitation wavelength enhancement, the FL signal is red-shifted with a simultaneous decrease in FL intensity, illustrating the excitation-dependent nature of the emission. The QYs of CDs derived from lemon/onion, date kernel, *sargassum fluitans*, and neem leaf were 23.6%, 12.5%, 18.2%, and 27.2%, respectively.

Furthermore, several plant part-derived CDs exhibited their highest emission wavelength with an excitation wavelength between 350 and 360 nm. The CDs from *Lycii fructus* [62], ginkgo leaf [66], and waste tea [70] had an excitation wavelength of 350 nm for their highest emission intensities at 430, 436, and 445 nm, respectively. For *Lycii fructus* CDs, the optimum emission peak center red-shifted from 415 to 545 nm upon excitation between 300 and 500 nm. The emission intensities enhanced when changing the excitation wavelength from 300 to 350 nm, and then lowered as the excitation wavelength enhanced. The maximum emission wavelength of the tea CDs moved from 440 to 460 nm with the enhancement of the excitation wavelength from 290 to 400 nm. The QYs of the CDs were 17.2%, 22.8%, and 7.1% for these three CDs, respectively. Next, the CDs from watermelon juice [65] and *Bauhinia* flower [77] showed an emission wavelength at 439 and 442 nm with excitation at 355 nm. The emission peak of CDs from watermelon juice progressively changed from 424 to 512 nm when enhancing the excitation wavelength from 315 to 455 nm, demonstrating the excitation-dependent nature of the emission. The FL emission peak of CDs from the *Bauhinia* flower progressively red-shifted with an increase of excitation wavelength from 295 to 385 nm, showing the excitation-dependent character of the emission. The QYs were 10.6% and 27% for the two above CDs. For CDs from henna leaf [67], mint leaf [71], groundnut [54], and *Lantana camara* berry [61], the optimum emission spectra were at 440, 441, 443, and 450 nm, respectively, with a 360 nm excitation wavelength. All of these CDs also displayed excitation-dependent emission with red-shift behavior. The FL spectrum of henna leaf-CDs using an excitation wavelength of 360 nm showed a shoulder peak at 500 nm. The signal is correlated to the surface state emission that enhanced the width of the FL spectrum. The dual FL bands were monitored in relation to core and surface state emission [127]. The primary source of FL was correlated to the core emission (at 440 nm), which was smaller than that of surface states. The QYs of the CDs were 28.7%, 7.64%, 7.87%, and 33.15% using the carbon sources of the henna leaf, mint leaf, groundnut, and *Lantana camara* berry, respectively.

Moreover, several plant part-derived CDs showed the highest emission spectra with an excitation wavelength of between 370 nm to 390 nm. Tomato juice [57], papaya flesh [58], mushroom [50], and water chestnut/onion [56] CDs exhibited their highest emission peaks at 440, 450, 455, and 475 nm at an excitation wavelength of 370 nm, with the QYs of 13.9%, 18.98/18.39%, 15.3%, and 12%, respectively. The emission peak of tomato juice CDs red-shifted and the FL intensity lowered when the excitation wavelength was increased from 350 to 410 nm. The tomato juice-derived CDs also exhibited the optimum upconverted emission peak at 470 nm at an excitation wavelength of 780 nm, pointing out their infrared upconversion nature, probably due to anti-Stokes FL [128]. The FL spectra show the typical nature of papaya powder-CDs where their emission peak shifted from 445 to 530 nm by changing the excitation wavelength from 350 to 490 nm. The CDs from mushroom exhibited an emission peak with red-shift behavior, and their FL intensity lowered when the excitation wavelength was increased from 370 to 450 nm. Moreover, pineapple peel [64], rose flower [79], and coconut water [81] produced CDs with the optimum emission wavelengths of 435, 435, and 450 nm with excitation wavelengths of 380, 390, and 390 nm, respectively. While altering the excitation wavelength, the peak position was red-shifted and the peak intensity decreased. They produced a QY of 42%, 13.45%, and 2.8%, respectively.

Some plant part-derived CDs have a longer excitation wavelength at and above 400 nm, such as CDs from saffron (400 nm) [52], water hyacinth (400 nm) [86], white pepper (420 nm) [74], lemon juice (420 nm) [59], and aloe (441 nm) [48] with optimum emission wavelengths at 485, 486, 520/668, 540, and 503 nm, respectively. The FL QYs were 23.6%, 27%, 10.4%, 21%, and 10.37% for these CDs. The emission peak red-shifted as the excitation wavelength was enhanced from 340 to 440 nm, 280 to 540 nm, 360 to 520 nm, and 443 to 525 nm for saffron, water hyacinth, lemon juice, and aloe CDs, respectively. On the other hand, white pepper CDs showed two emission peaks at 520 and 668 nm. The intensities at these wavelengths enhanced with increases in the the excitation wavelength ranging from 400 to 420 nm, and then lowered as the excitation wavelength was increased to 440 nm. The emission at 520 nm was red-shifted dependent on excitation from 400 to 440 nm, whereas the emission at 668 nm showed excitation-independent character as the excitation shifted from 400 to 420 nm and subsequently blue-shifted with the excitation from 420 to 440 nm. The emission peak at 668 nm was from porphyrin derivatives, suggesting that these porphyrin derivatives combine with CDs.

Three CDs derived from pineapple, muskmelon, and tomato exhibited three different excitation wavelengths. Pineapple CDs exhibited excitations at 325, 417, and 425 nm with the highest emissions at 438, 516, and 543 nm, respectively [94]. Muskmelon CDs showed emission peaks at 432, 515, and 554 nm by excitation at 342, 415, and 425 nm, respectively [92]. Tomato CDs showed emission peaks at 450, 520, and 560 nm by excitation at 360, 420, and 460 nm, respectively [93]. These three excitation peaks and emission peaks were for blue, green, and yellow CDs. The emission intensities of three CDs from pineapple progressively changed from 439 to 494 nm, 517 to 553 nm, and 528 to 563 nm with enhancing excitation wavelengths ranging from 300 to 420 nm, 350 to 450 nm, and 350 to 470 nm, respectively. Muskmelon-derived CDs were examined for emission spectra excited from 300 to 410 nm, 320 to 460 nm, and 350 to 470 nm for their three CDs while tomato CDs were excited from 310 to 410 nm, 380 to 480 nm, and 390 to 500 nm. These findings indicated excitation-dependent emission behavior. The QYs of the CDs were 18.0, 37.6, and 44.7%, for pineapple CDs; 7.07, 26.9, and 14.3%, for muskmelon CDs; and 12.70%, 4.21%, and 2.76%, for tomato CDs. Moreover, CDs derived from *Prosopis juliflora* showed two emission peaks at 396 and 437 nm upon excitation at 325 and 350 nm, revealing two kinds of transition with a QY of 5% [83]. In the last example, CDs from *Citrus sinensis* showed a maximum FL emission at 455 nm excited at 365 nm, while CDs from *Citrus limon* showed two maximum emission peaks at 390 and 435 nm when excited at 330 nm. The QYs of CDs were estimated as high as 16.8% and 15.5% for *Citrus sinensis* and *Citrus limon*, respectively. Quinine sulfate, rhodamine 6G, and fluorescein sodium were used as references to calculate all the above QYs. A summary of the FL properties including excitation and emission wavelengths as well as QY is given in Table 3.

Excitation-dependent emission character is usually shown in CDs synthesized from natural precursors, demonstrating the presence of various particle sizes and surface defects [129]. Red shift is one of the most important characters of CDs, maybe because of their distinct particle sizes and surface energy trap distributions [130]. Size-dependent FL emission is primarily due to quantum confinement; smaller size causes a larger band-gap [81]. The generation of surface defects produces complicated emissive traps on the CDs, inducing the excitons to give rise to emission during radiative recombination [131]. Multi-color emission occurs due to different sizes and the emissive trap sites’ distribution, such as -COOH groups [100]. The functional groups have numerous energy levels that generate a series of emissive traps on the CDs’ surface [111]. The CDs in question usually reported being low in FL QY. Until now, elemental doping has been utilized as a promising approach to tuning the intrinsic FL properties of CDs and is considered to produce highly FL QY [132]. The doping of CDs leads to FL enhancement and a shift in emission spectra [33]. Elemental doping can be categorized into heteroatoms (non-metals) and metal atoms [133]. The primary hindrance to metal atom doping is correlated with the increase of toxicity. Therefore, single-heteroatom dopants such as N, S, P, and B, and co-doping multiplex heteroatoms such as N with S, P, B, and others, are preferable as dopants for CDs, which can improve the QY [133,134]. Among them, N-doped CDs, which are derived from N-rich precursors such as cheap and safe proteins, amino acids, and other N-containing natural precursors, are more interesting because of their superior ability to increase FL QY [135]. Several plant part-derived CDs have high QY after the addition of N-containing compounds such as EDA and NH_3_ as discussed in the synthesis section. The presence of electron-rich N-, P-, and S-containing functional groups or N, S, and P doping is regularly recognized as the cause of superior auxochromes resulting in a high QY.

## 4. Biosensing Applications of Plant Part-Derived CDs

Plant part-derived CDs have been utilized as biosensors to detect bioactive molecules such as amino acids, thiols, vitamins, enzymes, bacteria, biological pigments, nucleic acids, and proteins (Table 4), and several important molecules such as metal ions, dyes, pesticides, drugs, NO_2_^−^, and borax in biological samples (biofluids and foods) (Table 5). In this section, we overview the biosensing applications of plant part-derived CDs based on target categories.

### 4.1. Sensing of Amino Acids and Thiols

Vandarkuzhali et al. applied pineapple peel CDs as “turn-off” and “turn-on” FL sensors toward Hg^2+^ and Cys, respectively [64]. They designed individual elementary logic operations such as NOT and IMP gates based on spectral changes, using CDs as a probe and Hg^2+^ and Cys as chemical inputs. The emission spectra of the CD/Hg^2+^ complex with various amino acids including Ala, Lys, Asp, Arg, Met, Pro, Thr, Trp, His, Val, Phe, Ser, and Cys were recorded and the results clearly indicated that other than Cys, amino acids did not show any FL changes. Emission intensity increased in the presence of Cys. The limit of detection (LOD) of Cys via the CD/Hg^2+^ system was measured as 0.87 µM. Lu et al. used watermelon juice-derived CDs for selective and sensitive FL sensors towards Cys in an N-CD/Fe^3+^ system via the “turn-on” effect [65]. The FL of the N-CD/Fe^3+^ system was restored after adding Cys. For selectivity, the alterations in the FL intensity of the N-CD/Fe^3+^ system induced by adding other amino acids such as Gly, Lys, Ala, Arg, and Thr were examined. The FL spectra of the N-CD/Fe^3+^ system after adding diverse amino acids exhibited an insignificant effect on the FL of the N-CD/Fe^3+^ system, showing substantial selectivity towards Cys. The competitive combination between N-CDs and the thiol group in Cys toward Fe^3+^ ions resulted in releasing Fe^3+^ ions from the N-CDs’ surface, and thus the FL was recovered. Good linearity of Cys was achieved with the concentration of 0–250 μM with a correlation coefficient (R^2^) value of 0.9869 and LOD of 0.27 μM. Additionally, Chen et al. applied tea CDs for sensing Cys through the systems of tea CD/CrO_4_^2−^ and tea CD/Fe^3+^ [70]. The FL of the tea CDs could be reduced by CrO_4_^2−^ and Fe^3+^ ions and recovered by Cys. CrO_4_^2−^ ions could be transformed into Cr^3+^ by Cys, generating in the removal of the inner filter effect (IFE) and FL recovery for about 70.91% of tea CDs. Fe^3+^ ions interacted much more easily with Cys compared to -OH groups, thus the recovery of FL for the tea CDs was at about 60.23%. The FL intensity was no longer altered 1 min after adding Cys to the tea CDs. The FL of CD/ionic systems was increased progressively with enhancing Cys concentrations. The LODs of Cys were approximately 8.785 and 153.5 μM via the systems of tea CD/CrO_4_^2−^ and tea CD/Fe^3+^, respectively.

**Figure 6:**
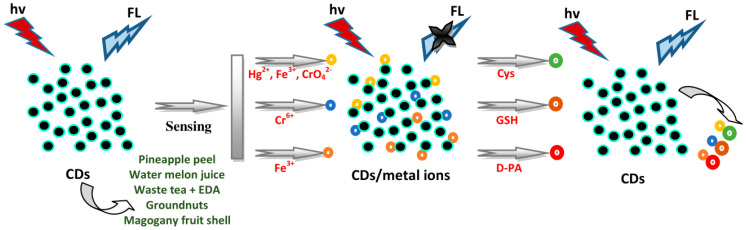
Illustration of the use of plant part-derived CDs for the detection of amino acids and thiols. “Turn-on” FL intensity was shown after the interaction of Cys, GSH, and D-PA with CD/ion systems. Abbreviations: GSH, glutathione; D-PA, D-penicillamine.

Furthermore, Roshni et al. used groundnut CDs to detect Cr^6+^ and glutathione (GSH) [54]. Cr^6+^ ions induced a reduction of the FL signal which could be restored using GSH, showing the “off-on” sensing effect. The thiol group of Cys can give electrons to other molecules, thereby showing great reducing nature in the reduced state. Upon giving an electron, GSH itself is reactive and immediately interacts with another reactive GSH to form glutathione disulphide (GSSG). The removal of Cr^6+^ by GSH is proper to the aspects of green chemistry. Gunjal et al. applied mahogany fruit shell CDs as dual-mode nanosensors for Fe^3+^ and D-penicillamine (D-PA) [90]. Fe^3+^ ions reduced the FL emission signals of CDs. D-PA with the thiol group has a high affinity for Fe^3+^, generating the recovery of FL intensity (76.4%) and decreasing the absorbance of the CD/Fe^3+^ system, forming the D-PA/Fe^3+^ complex and exhibiting a slight emission band shift. The binding of Fe^3+^ and CDs was disturbed and released the Fe^3+^ from the CDs’ surface with the addition of D-PA. The CD/Fe^3+^ system was able to select D-PA from among the other interfering substances such as thiourea, glucose, cystamine hydrochloride, dopamine (DA), GSH, D-tyrosine, glycine, urea, Ni^2+^, Ca^2+^, Na^+^, K^+^, Cl^−^, etc. Only D-PA enhanced the FL intensity of a CD/Fe^3+^ system whereas the others were incapable of generating a substantial effect on FL intensity. The analysis of D-PA with the concentration range of 0–322 µM showed an R^2^ of 0.9823 and LOD of 49.59 µM. The absorbance intensity of the CD/Fe^3+^ system was lowered by increasing the D-PA concentration, with an obvious change in the absorption wavelength. Linearity was achieved within the concentrations from 0 to 268 µM with an R^2^ of 0.989 and LOD of 39.27 µM. Hence, the CD/Fe^3+^-based FL and UV-vis probes acted as a selective probe for detecting D-PA. Based on the above results, a schematic illustration of plant part-derived CDs for the sensing of amino acids and thiols is illustrated in Figure 6.

### 4.2. Sensing of Vitamins

Ascorbic acid (AA) or vitamin C was analyzed using several CDs. Yadav et al. utilized CDs from *Azadirachta indica* leaves for the detection of AA [69]. The oxidized 3,3’,5,5’-tetramethylbenzidine (ox-TMB) with a blue color gives a notable platform for the detection of reductant molecules. The FL emission intensity of N-CDs was only slightly decreased in the presence of TMB (N-CDs + TMB); however, emission intensity was fully reduced in the presence of H_2_O_2_ (N-CDs + TMB + H_2_O_2_), indicating complete TMB oxidation. However, ox-TMB is reduced to TMB in the presence of AA (N-CDs + TMB + H_2_O_2_ + AA). A UV-vis sensitivity trial was performed by introducing various AA concentrations (0–120 μM) into ox-TMB. The absorbance at 652 nm was lowered and the color was altered from blue to colorless upon incrementation of the AA concentration. For the selectivity test, various reducing agents such as KBr, KI, cinnamic acid (CNA), glucose, CA, benzoic acid (BZA), oxalic acid (OA), adipic acid (ADA), tartaric acid (TTA), uric acid (UA), and DA were mixed into the ox-TMB solution. There were negligible changes in the absorption of ox-TMB in the presence of these reducing agents. The AA-dependent reduction of ox-TMB exhibited consistent linearity from 5 to 40 μM AA with an R^2^ of 0.998 and LOD of 1.773 μM. The AA sensing on the peroxidase mimetic catalytic activity of N-CDs by the oxido-reduction of TMB was fully color-dependent. Moreover, Candra et al. used mustard seed CDs for the colorimetric detection of AA [55]. AA induced the reduction of ox-TMB to native TMB. For selectivity, other common reducing agents (UA, CNA, TTA, CA, GSH, OA, ADA, BZA, and tannic acid, TNA) were added into a blue-colored ox-TMB solution. There was negligible change in ox-TMB absorbance at 652 nm in the presence of the reducing agents except GSH. However, in the presence of GSH, the absorbance of ox-TMB decreased insignificantly compared to AA. The valuable decrease in absorbance with a color change from blue to colorless in the presence of AA indicated the high reduction ability of AA compared to GSH. Hence, the suggested method was selective for the colorimetric detection of AA. A UV-vis titration trial was performed upon adding various AA concentrations to the ox-TMB. The absorption of ox-TMB lowered with increases of the AA concentration range of 10–70 μM. CDs exhibited a useful probe for detecting AA selectively and sensitively with an R^2^ of 0.9949 and LOD of 3.26 μM. Furthermore, Chen et al. applied tea CDs to sense AA [70]. The FL intensity of both tea CD/CrO_4_^2−^ and tea CD/Fe^3+^ systems increased dramatically with enhanced AA concentrations. The recoveries of AA for the tea CD/CrO_4_^2−^ and tea CD/Fe^3+^ systems were 78.07% and 55.32%, respectively. A good linear relationship was observed with the LOD of 87.02 μM for the tea CD/CrO_4_^2−^ system and 19.78 μM for the tea-CD/Fe^3+^ system. Therefore, both systems can potentially be employed to detect AA. Moreover, Raveendran et al. utilized mint leaf CDs to detect AA [71]. The FL reduction of CDs by Fe^3+^ ions can be recovered by particular biomolecules. The CD/Fe^3+^ system acted as an efficient sensor for detecting AA. The selective detection of AA by the system from other biomolecules such as glucose, urea, Cys, Gly, Met, Glu, and CA was examined, and it was found that only AA can significantly restore FL intensity, indicating that the FL probe was selective for AA. By observing the recovered FL of CDs, it is simple to detect AA. The FL recovery of the CD/Fe^3+^ system enhanced in a linear fashion with increases in AA concentration, with an R^2^ of 0.985 and LOD of 0.079 µM [71].

**Figure 7:**
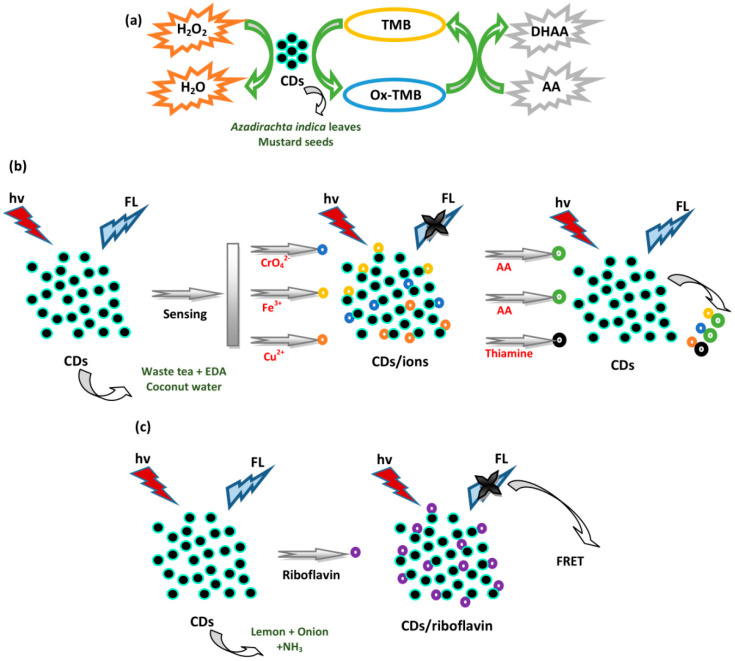
Schematic illustrations of plant part-derived CDs for detecting AA, thiamine, and riboflavin. (**a**) The TMB oxidation and colorimetric detection of AA. (**b**) The “turn-on” effect on the FL intensity of CDs for the detection of AA and thiamine. (**c**) The FL-quenching behavior of CDs was observed after interacting the CDs with riboflavin through the FRET system. Abbreviations: AA, ascorbic acid; TMB, 3,3′,5,5′-tetramethylbenzidine; FRET, fluorescent resonance energy transfer.

Thiamine and riboflavin are well known as vitamin B1 and vitamin B2. Each vitamin was analyzed by different research groups. Purbia and Paria used coconut water CDs to sense thiamine [81]. The CDs were examined for a simple “turn-on” FL probe in detecting thiamine. The FL emission intensity of CDs was quenched after adding Cu^2+^ and then turned on after adding thiamine. Selective detection was also examined in the presence of several molecules such as Mg^2+^, Ca^2+^, K^+^, Na^+^, Cl^−^, NO_3_^−^, and SO_4_^2−^, which are present in urine and blood serum and obtained an insignificant effect in the detection. The enhancement of the FL emission intensity of CD/Cu^2+^ in the presence of thiamine exhibits linearity in the concentration range of 10–50 µM with the LOD of 0.28 µM. Finally, Monte-Filho et al. used CDs derived from lemon and onion juices for the detection of riboflavin [78]. FL resonance energy transfer (FRET) between the riboflavin as acceptor and the CDs as the donor occurred. The selectivity of the CDs for the detection of riboflavin was also tested for several substances such as folic acid (FA), CA, AA, biotin, pantothenic acid, niacin, cyanocobalamin, sucrose, fructose, D-glucose, thiamine, Cu^2+^, Zn^2+^, Fe^2+^, Fe^3+^, Mg^2+^, Ca^2+^, Na^+^, and K^+^. The substances did not indicate a valuable change in the FL emission intensity of the system, showing that the system was selective for riboflavin. Linearity was achieved with the riboflavin concentration of 0.27–7.97 µM, showing a good analytical method for detecting riboflavin. The LOD and RSD were 0.003 µM and <2.6% (*n* = 3), respectively. Schematic illustrations of the sensing applications for detecting AA, thiamine, and riboflavin discussed above are represented in Figure 7.

### 4.3. Sensing of Enzymes

Long et al. applied CDs derived from white pepper as a “turn-on” FL sensor to detect coenzyme A (CoA) with the help of Cu^2+^, where the emission at 668 nm hardly shifted and acted as standard [74]. Different conditions influencing CD/Cu^2+^ complex formation such as incubation pH, the concentrations of the CDs and Cu^2+^, and reaction time were probed to reach high sensitivity for the detection of CoA. CDs exhibited higher stability and FL response, and the CD/Cu^2+^ system showed a decrease in FL intensity, when 2 mg mL^−1^ CDs, 65 μM Cu^2+^, pH 7.4, and 15 min reaction time were selected. The addition of CoA recovered the reduced FL in the CD/Cu^2+^ system within 5 min. The nature of the selection of CoA by the CD/Cu^2+^ system was analyzed by introducing several substances including DA, GSH, Hcy, Cys, Leu, Trp, Tyr, Met, Ser, Thr, Ile, Val, Ala, Phe, and Lys, showing an insignificant effect on the FL intensity of the system. The peak ratio (F_520_/F_668_) exhibited good linearity with the CoA concentration of 0–150 μM with R^2^ of 0.995 and LOD of 0.00875 μM. The determination of CoA was also investigated by Hu et al. [56]. They used S- and N-CDs derived from water chestnut and onion as an “off-on” FL sensor to detect CoA. The -COOH groups on the S- and N-CDs’ surface were able to bind with Cu^2+^ ions, reducing the FL of these S- and N-CDs. CoA then recovered the FL of the S- and N-CDs. To assess the selectivity for CoA, several substances, including biothiols (Cys, Hcy, and GSH), anions, amino acids, glucose, lactose, sucrose, guanine, UA, DA, adenosine, and adenosine triphosphate (ATP), were investigated for FL response. The glucose, anions, lactose, sucrose, DA, guanine, ATP, adenosine, UA, and most amino acids did not influence the detection of CoA. Biothiols could only partially restore the FL, but to a degree lower than that of CoA. The pKa value of Cys (8.00), Hcy (8.87), and GSH (9.20) was higher than that of CoA as acid (6.40). This method can be utilized for the particular determination of CoA compared to the biothiols at pH 5.8. The thiolate of CoA interacts more easily with Cu^2+^ ions than Cys, Hcy, and GSH at pH 5.8. Linearity was achieved for CoA detection with the concentration of 0.03–40 μM, with the LOD of 0.01 μM.

**Figure 8:**
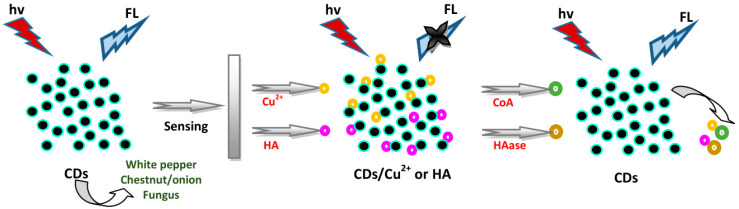
Illustration of plant part-derived CDs for detecting enzymes. The “turn-on” effect on emission intensity was exhibited after adding CoA and HAase to CD/Cu^2+^ and CD/HA systems, respectively. Abbreviations: CoA, coenzyme A; HAase, hyaluronidase; HA, hyaluronic acid.

Furthermore, Yang et al. utilized fungus CDs to detect hyaluronic acid (HA) and hyaluronidase (HAase) [50]. The CDs showed electrostatic adsorption for HA, generating FL quenching via the aggregations. Enzymatic digestion between HA and HAase took place; therefore, FL recovery occurred upon introducing HAase. The FL intensities showed insignificant change with the addition of HAase, deoxyribonuclease (DNase) I, papain, Mn^2+^, Zn^2+^, Pb^2+^, Ba^2+^, and Co^2+^ to the CD solution, showing the selectivity of the CDs for HA. Also, the FL exhibited negligible change when adding DNase I, ribonuclease (RNase) I, exonuclease (EXO) I, papain, Zn^2+^, Co^2+^, Ba^2+^, and Mn^2+^ to the CD/HA system, indicating that the CD/HA system was also selective to HAase. Thereby, linearity was achieved with HAase concentrations ranging from 0.2 U to 10,000 U mL^−1^ and with HA concentrations ranging from 50 pM to 50 µM. The FL intensity of the CDs decreased with enhancing HA concentrations from 50 pM to 50 µM with an R^2^ of 0.995 and LOD of 0.3 × 10^−5^ µM. The FL of the CDs was recovered with increasing HAase concentrations from 0.2 to 1000 U mL^−1^ with an R^2^ of 0.994 and LOD of 0.1 U mL^−1^. A schematic illustration of the above CDs for detecting CoA and HAase is depicted in Figure 8.

### 4.4. Sensing of Bacteria

**Figure 9:**
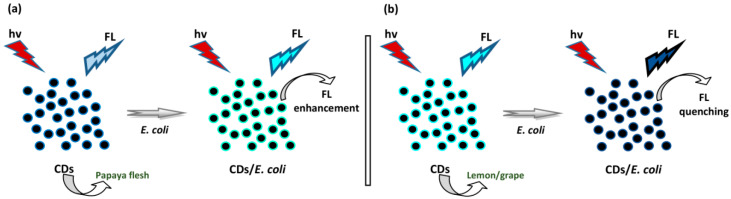
Schematic illustration of plant part-derived CDs for the sensing of *E. coli* with (**a**) FL enhancement and (**b**) FL quenching. Different effects on the FL emission intensity of CDs were observed after adding *E. coli* into CD solution.

Wang et al. detected *Escherichia coli* (*E. coli*) by using CDs from papaya in water (W) [58]. W-CDs were used as a method expected to sense *E. coli*. *E. coli* induced a notable incremental impact on the FL emission of W-CDs (Figure 9a). Linearity was reached in concentrations ranging from 10^5^ to 10^8^ cfu mL^−1^ of *E. coli* with a LOD of 9.5 × 10^4^ cfu mL^−1^. On the contrary, the addition of *E. coli* with similar concentrations induced an unresponsive effect on the FL of ethanol (E)-CDs. Therefore, E-CDs are incapable of detecting *E. coli*. CDs reached an aqueous medium containing mannose, showing strong interaction with FimH (a two-domain protein). The FimH proteins on the tip of the fimbriae of *E.coli* bind with mannose in the W-CDs to generate the FL shift of W-CDs. Ahmadian-Fard-Fini and coworkers also detected *E. coli* based on the FL sensor of CDs derived from lemon and grapefruit extracts [60]. The FL signals of various concentrations of CDs labeled with *E. coli* were observed. The quantity of unknown concentrations of *E. coli* was able to be measured as well. The addition of *E. coli* to CD solution induced FL quenching (Figure 9b). The quantitative detection of *E. coli* was achieved using the Stern–Volmer relationship. A standard curve was achieved in concentrations ranging from 0 to 9 × 10^7^ cfu mL^−1^.

### 4.5. Sensing of Bio-Pigments

**Figure 10:**
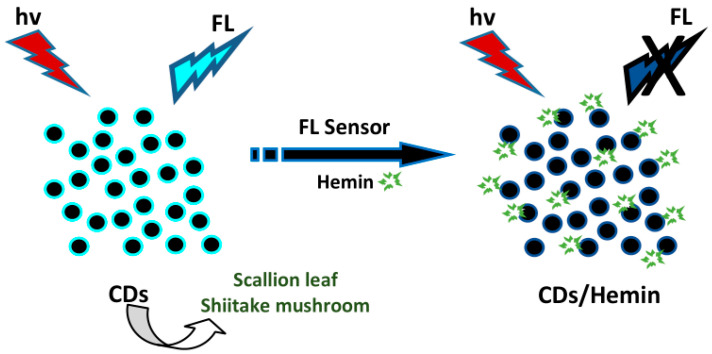
Schematic illustration of plant part-derived CDs for the sensing of hemin. The addition of hemin to CD solution exhibited an FL quenching effect.

Zhang et al. used N- and S-CDs derived from scallion leaves to sense hemin [68]. The FL intensity of the N- and S-CDs was reduced when increasing the hemin concentration from 0.5 to 10 µM. The FL quenching mechanism originated from the IFE and photoinduced electron transfer (PET). To examine selectivity for hemin, several substances including amino acids (Arg, Thr, Asn, Leu, His, Gln, Phe, Gly, Ala, Pro, Ile, Met, Val, Lys, Ser, Trp, Asp, Glu, and Tyr) and several metal ions (Fe^3+^, Cd^2+^, Mg^2+^, Zn^2+^, Fe^2+^, Al^3+^, Cr^2+^, Co^2+^, Cu^2+^, Ag^+^, Hg^2+^, Mn^2+^, and Pb^2+^) were also studied. Compared to amino acids and metal ions, hemin provided typical FL quenching for the CDs, showing that the sensor has a high selectivity for hemin. The FL intensity of the N- and S-CDs quenched to a steady value in the presence of hemin. The hemin sensor showed linearity ranging from 0.5 to 10 µM and a LOD of 0.1 µM. Wang et al. also detected hemin by using CDs derived from dehydrated shiitake mushroom [51]. As a natural metalloporphyrin, hemin molecules have intrinsic peroxidase-mimicking activity and could interact with the CDs’ surface via electrostatic and π-π stacking interactions; therefore CDs, tend to assemble into complexes and quench FL intensity through electron or energy transfer. The FL emission of CDs quenched notably with increases in hemin concentration. The selectivity assay for the hemin was evaluated by introducing several ionic species (Fe^3+^, Al^3+^, Ca^2+^, Zn^2+^, Cu^2+^, Mg^2+^, Na^+^, K^+^, Cl^−^, PO_4_^3−^, CO_3_^2−^, SO_4_^2−^, and NO_3_^−^) and protein species (histone, Hb, cyt-c, GSH, BSA, AA, Glu, Cys, Gly, Phe, and Ser). These substances exhibited no clear interruption in the detection of hemin. The FL quenching was linear with hemin concentrations from 0.4 to 8.0 µM with an R^2^ of 0.9987 and an LOD of 0.12 µM, along with a relative standard deviation (RSD) of 3.8% at 2.0 µM hemin. The illustration of plant part-derived CDs’ sensing application for detecting hemin is depicted in Figure 10.

### 4.6. Sensing of Nucleic Acids and Proteins

Godavarthi et al. utilized N-CDs derived from *Sargassum fluitans* to detect DNA [49]. The FL-tagging capabilities of N-CDs with nucleic acids were exhibited utilizing gel electrophoresis. A notable enhancement in FL properties was found when tagging N-CDs with nucleic acids, and this particular case contributes to the visualization of these nucleic acids. Three nucleic acids including double-stranded DNA, single-stranded DNA, and RNA exhibited identical cases when tagging with N-CDs. Therefore, N-CDs can be utilized as an optional fluorophore towards poisonous organic staining agents in visualizing nucleic acids. N-CDs without DNA exhibited no FL, but N-CDs tagged with DNA exhibited an FL band. The FL intensity of N-CDs was enhanced primarily in the presence of DNA. The increased FL of the N-CD/DNA mixture was generated from the combination of DNA bases and the passivated functional groups of N-CDs. The lone pairs of an electron from nitrogen and oxygen in passivated N-CDs interacted with the DNA bases through H-bonds to visualize the DNA. Various concentrations of N-CDs (15.15–1515 µM) were tagged in all wells of double-stranded DNA. From the observation, even 15.15 µM is enough for the appropriate visualization of DNA.

**Figure 11:**
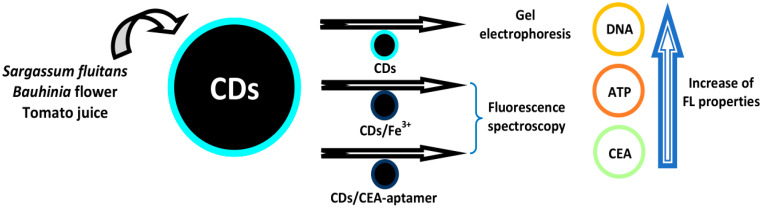
Schematic illustration of plant part-derived CDs for the sensing of nucleic acids and proteins. The excellent FL-tagging capabilities of N-CDs with nucleic acids were proved using gel electrophoresis. The enhancements in CD/Fe^3+^ and CD/CEA-aptamer were shown after interacting with ATP and CEA, respectively. Abbreviations: CEA, carcinoembryonic antigen; ATP, adenosine triphosphate.

Huang et al. applied *Bauhinia* flower-derived N-CDs as a “turn-on” FL probe to determine ATP [77]. The FL of the N-CD/Fe^3+^ system could be restored after adding ATP because of its highest affinity for Fe^3+^ via Fe-O-P bonds. ATP could act as a complexing agent for Fe^3+^ ions. For the selectivity test, other biomolecules including cytosine 5′-triphosphate (CTP), guanosine 5′-triphosphate (GTP), uridine 5′-triphosphate (UTP), UA, AA, and DA were examined in the system. Only ATP could recover the FL intensity, while nearly all other substances changed the FL intensity insignficantly. The findings showed that this system had good selectivity towards ATP. The FL intensity of the N-CD/Fe^3+^ system was recovered with increases in ATP concentration. A linear relationship was achieved with ATP concentration from 0.01 to 450 μM, with an R^2^ of 0.9978 and LOD of 0.005 μM. Furthermore, Miao and coworkers investigated label-free FL detection of carcinoembryonic antigen (CEA) and CEA-aptamer utilizing CDs derived from tomato juice [57]. The plentiful -COOH groups of the CDs allowed high single-stranded DNA (ssDNA) adsorption to the CDs’ surface via π-π stacking interactions, generating FL quenching by the formation of the CD-aptamer complex. CEA-aptamers were added to produce signals relating to targets, and the assembly was generated by π-π stacking between CDs and aptamers. The addition of aptamer resulted in a 60% quenching of the FL, indicating that CEA-aptamer combined with CDs and reduced their FL. For the selectivity study, several proteins such as CA125, CA15-3, BSA, AFP, DA, thrombin, glucose oxidase, and tyrosinase were investigated with the presence of CEA. CEA was obviously differentiated from others, suggesting that this method possessed good selectivity. Besides, numerous substances such as Vitamin B1, Vitamin B2, AA, norepinephrine, glucose, FA, bile acid, insulin, lactose, Fe^3+^, Ca^2+^, Na^+^, and K^+^ were introduced into this detection method. These biomolecules and ions exhibited insignificant effects on the FL intensity of the probe. Under the reaction of CDs with the aptamers, the FL intensity of CDs was quenched according to the increased concentrations of CEA-aptamer ranging from 0.00565 to 50 μM with an R^2^ of 0.989 and the LOD of 0.00188 µM. The higher binding affinity between CEA-aptamer and CEA than in the π-π stacking interactions was used to obtain the FL recovery of CDs once the CEA was introduced. The analysis of CEA concentrations ranging from 1 ng mL^−1^ to 500 µg.mL^−1^ had an R^2^ of 0.974 and LOD of 0.3 ng.mL^−1^. The schematic illustration of plant part-derived CDs for detecting nucleic acids and proteins is depicted in Figure 11. The summary of the analytical detections of all the above-mentioned biosensors based on plant part-derived CDs in detecting bioactive molecules can be viewed in Table 4.

### 4.7. Sensing of Metal Ions

Bandi et al. utilized N-CDs from *Lantana camara* berries to detect Pb^2+^ ions in body fluids [61]. The FL response of N-CDs for several metal ions including Cr^3+^, Al^3+^, Fe^3+^, Cd^2+^, Ni^2+^, Hg^2+^, Mn^2+^, Zn^2+^, Mg^2+^, Pb^2+^, Fe^2+^, Ba^2+^, Ca^2+^, Cu^2+^, Sn^2+^, Na^+^, and K^+^ was evaluated to assess their ability to interact with CDs. Among several metal ions, only Pb^2+^ induced valuable FL quenching, indicating that the N-CDs could be used as a “turn-off” sensor to selectively detect Pb^2+^. N-CDs exhibited a linear response towards Pb^2+^ ions in concentrations of 0–0.2 µM with an R^2^ of 0.998 and LOD of 0.00964 µM. N-CDs were employed to determine Pb^2+^ ions in human serum and urine samples. The different concentrations of Pb^2+^ ions were spiked to the samples. The recoveries were between 98.40 and 102.10% with RSD values of <2%, showing the potential of this sensing system in the detecting of Pb^2+^ in biological samples. Desai and coworkers detected Hg^2+^ ions using muskmelon fruit (*Cucumis melo*) CDs in human serum samples [92]. The FL intensity of green CM CDs at 515 nm was more greatly reduced than that of blue and yellow CM CDs upon adding Hg^2+^ ions. For the selectivity study, the quenching efficiency of Hg^2+^ in the presence of other species including metal ions (Cr^3+^, As^3+^, Co^2+^, Cu^2+^, and Ni^2+^), anions (PO_4_^3−^, Cr_2_O_7_^2−^, S^2−^, Cl^−^, NO_3_^−^, and CH_3_COO^−^), and pesticides (triazophos, thiram, quinalphos, monocrotophos, and chlopropham) was evaluated. An insignificant change was shown in the FL intensity of green CMCDs with the other species mixtures. Hence, the emission intensity of green CM CDs was reduced only in the presence of Hg^2+^, showing that the green-CMCDs-based FL “turn-off” method could be used to detect Hg^2+^. Green CM CDs served as a probe in detecting Hg^2+^ ions with broad linearity with the concentration range of 1–25 μM with an R^2^ of 0.9855 and LOD of 0.33 μM. The recoveries of Hg^2+^ ions in human serum samples were achieved ranging from 96.88% to 100.89% with an RSD of 0.13–1.04%. Hoan et al. used lemon CDs to detect V^5+^ ions in fetal bovine serum samples based on FL quenching [59]. For the selectivity test, the FL intensity alteration of CDs in the presence of metal ions (Zr^4+^, Fe^3+^, Er^3+^, Ca^2+^, Fe^2+^, Co^2+^, Ni^2+^, Mn^2+^, Sr^2+^, Mg^2+^, Na^+^, K^+^, and Ag^+^) and mixtures of metal ions and V^5+^ were investigated. The FL intensity of the mixture of metal ions and CDs was quenched notably in the presence of V^5+^. Hence, insignificant decreases in FL intensity were observed after adding metal ions into the CDs. These findings showed that CDs are selective to V^5+^. The FL intensity of the CDs reduced significantly upon enhancing the concentration of V^5+^ ions. The selectivity experiments confirmed that this FL sensor was specific to V^5+^ ions with an LOD of 27.36 µM. Further, FL sensing for V^5+^ ions was employed in fetal bovine serum samples. V^5+^ ions can successively reduce the FL intensity of the CDs in blood serum samples. The FL intensity ratio showed linearity with the V^5+^ concentration in serum samples and the FL quenching received a saturation point at 854.8 µM of V^5+^ ions in serum.

Furthermore, Sun et al. used *Lycii fructus*-derived CDs to detect Fe^3+^ ions in urine samples [62]. The FL intensity of the CDs was notably reduced by Fe^3+^ ions. For the selectivity study, various metal ions such as Cr^3+^, Fe^3+^, Al^3+^, Cd^2+^, Mn^2+^, Ca^2+^, Fe^2+^, Zn^2+^, Mg^2+^, Co^2+^, Cu^2+^, Hg^2+^, Pb^2+^, K^+^, Li^+^, and Na^+^ were introduced into the CD solution. Fe^3+^ had the ability to reduce the FL intensity of the CDs, whereas other metal ions had insignificant quenching effects. The CDs showed a good linearity response for Fe^3+^ ions with the concentration of 0–30 μM and an LOD of 0.021 µM. The CDs were employed to determine Fe^3+^ ions in the urine samples. The recovery of urine samples was 96.8–102.5% and the RSD ranged from 0.86% to 1.14%. Kailasa and coworkers used three tomato CDs to detect Fe^3+^ ions in biofluids [93]. For selectivity, a mixture of metal ions (Al^3+^, Ni^2+^, Ca^2+^, Zn^2+^, Ba^2+^, Hg^2+^, Cu^2+^, and Fe^2+^) and anions (PO_4_^3−^, S^2−^, Cr_2_O_7_^2−^, SO_4_^2−^, Cl^−^, F^−^, Br^−^, and I^−^) did not exhibit any valuable effect on the FL emission of three CDs, while the Fe^3+^ ion notably reduced the FL emission intensity. The selectivity was also investigated in the presence of organic components and biomolecules (glucose, fructose, urea, GSH, BSA, and AA) and amino acids (Phe, Thr, Val, Met, Trp, His, Tyr, Gln, and Cys), respectively. No obvious quenching was observed in the emission peaks by adding the above molecules. Upon adding Fe^3+^ ions, the FL intensity of the CDs decreased gradually in concentrations ranging from 0.1 to 2.0 μM, exhibiting a good linearity in with Fe^3+^ concentrations from 0.1 to 1.25 μM, with an R^2^ of 0.9581, 0.8397, and 0.9520, and LODs of 0.016, 0.072, and 0.065 μM using blue, green, and yellow CDs, respectively. Then, the applications for detecting Fe^3+^ ions in urine and plasma samples were explored. The recoveries were in the range of 98.40–99.00% and 95.80–97.90% for urine and plasma samples, with RSD below 1.2%.

**Figure 12:**
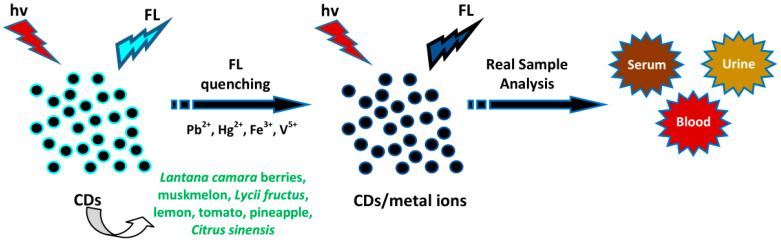
Schematic illustration of plant part-derived CDs for the sensing of various metal ions in biological samples. The addition of different metal cations produced a quenching effect on the FL intensity of CDs. Real sample analysis was performed to detect metal cations in biological samples.

Anibhai et al. also checked the performance of three (blue, green, and yellow) pineapple CDs in detecting Fe^3+^ ions in biofluids [94]. The FL intensity of blue CDs was significantly reduced by Fe^3+^ ions, indicating the formation of a blue CD/Fe^3+^ complex. To evaluate its selectivity, several chemical species such as metal ions (Al^3+^, Ni^2+^, Cu^2+^, Ca^2+^, and Zn^2+^), anions (PO_4_^3−^, Cr_2_O_7_^2−^, SO_4_^2−^, Cl^−^, and Br^−^), and pesticides (imidacloprid, acetamiprid, chlorpropham, glyophosate, and tebuconazole) were evaluated with and without Fe^3+^ ions. The addition of a mixture of various species to blue CDs did not valuably reduce the emission intensity; it was reduced only with Fe^3+^ ions, suggesting the selectivity of blue CDs for Fe^3+^ ions. FL quenching showed a linearity with concentrations of Fe^3+^ ions of between 0.05 and 500 μM with over 50% quenching at 500 μM Fe^3+^. Good linearity was obtained in concentrations of 0.05–100 μM with an R^2^ of 0.9967 and LOD of 0.03 μM. Then, the analysis of Fe^3+^ ions was performed in urine and plasma samples. This method showed good recovery of 98.7–102.6% with an RSD of 0.27–2.18%. Moreover, Chatzimitakos et al. used CDs from *Citrus sinensis* as an FL sensor to detect Fe^3+^ ions in biological samples [85]. For the selectivity study, the FL emission quenching caused by several metal ions such as As^3+^, Sb^3+^, Cd^2+^, Ca^2+^, Pb^2+^, Co^2+^, Mn^2+^, Cu^2+^, Hg^2+^, Se^2+^, Cr^2+^, Ni^2+^, Ag^+^, Na^+^, K^+^, and tartrazine was examined. Only Hg^2+^ and Pb^2+^ caused FL quenching of up to 20% and 12%, respectively, and the rest of the metal ions were not capable of significantly reducing the FL of the CDs. The disturbance from Pb^2+^ and Hg^2+^ is insignificant since their concentrations in biological samples are less than that of Fe^3+^ ions. The lowering of the FL intensity showed linearity with Fe^3+^ concentrations of 0.01–1.0 μM, with an R^2^ of 0.998 and LOD of 0.003 µM. The RSD (*n* = 3) was 2.5% for intraday (reproducibility) and 3.2% for interday (repeatability) tests of 1.0 μM Fe^3+^. Good recoveries were received ranging from 94.0% to 98.0% and from 94.0% to 99.0% for Fe^3+^ in human urine and blood samples, respectively. Therefore, matrix effects were absent, showing to the application potency of this probe to detect Fe^3+^ in relevant matrices. A schematic illustration of the above-mentioned plant part-derived CDs for sensing various metal ions in biological samples is represented in Figure 12.

### 4.8. Sensing of Drugs

Some drugs showed “turn-on” action and several others showed “turn-off” action for the FL intensity of plant part-derived CDs. For the “turn-on” action, Amin and coworkers used N-CDs from date kernel as an FL sensor to detect the zoledronic acid (ZA) drug in human serum via an N-CD/Fe^3+^ system [63]. The FL intensity of N-CDs was highly reduced in the presence of Fe^3+^ ions because of the bonding between the Fe^3+^ ions and functional groups of N-CDs. To evaluate the selectivity, several cations, anions and biomolecules which are commonly present in blood serum such as Phe, AA, UA, Cys, GSH, BSA, Ca^2+^, SO_4_^2−^, and CH_3_COO^−^ were investigated in terms of their effect on the response of the N-CD/Fe^3+^. No obvious changes in the emission were observed after adding other molecules or ions even with higher concentrations than ZA. The FL status was turned on by adding ZA because of the phosphate groups in ZA and the functional groups of N-CDs were competing to interact with Fe^3+^ ions, generating the release of Fe^3+^ ions from the N-CDs’ surface. This FL probe showed good performance for the ZA probe with a linear range of 0.1–10.0 µM and an LOD of 0.04 µM. The detection of ZA showed with good recoveries (92.2–104.0%) in human serum samples. The RSD was less than 3.2% for 0.1 µM of ZA. Pourreza and Ghomi used CDs derived from *Prosopis juliflora* leaves for sensing the chemet drug in human serum samples [83]. This method was based on the “off–on” FL of CDs. The “off” FL behavior was viewed in the presence of various concentrations of Hg^2+^ that were specifically recovered after adding the chemet drug. The recovery of FL intensity was because of the high affinity of the chelating property of the chemet drug for Hg^2+^ with a good recovery. Linearity was observed in the concentration range of 0.014–0.123 µM chemet with an LOD of 0.0077 µM. The RSD of the FL probe for the chemet was 1.3% with recoveries of 98.0–101.0% in human serum samples.

For the “turn-off” action, Jiang et al. utilized N-CDs derived from ginkgo leaf for the sensitive determination of salazosulfapyridine (SASP) in mouse plasma because of the robust quenching effect of SASP [66]. The FL intensities of the N-CDs quenched upon increasing SASP concentration. The quenching efficiency was 84.68% using 200 µM SASP. For selectivity, the effects of sulfonamide, amoxicillin, aspirin, sinomenine, and levofloxacin on the FL of N-CDs were investigated. The other medications had insignificant effect on FL quenching, excluding SASP. Plasma contains various carbohydrates, proteins, amino acids, and minerals, and so furthermore, the effects of glucose, Cys, human serum albumin, urea, Fe^3+^, Mg^2+^, Ga^2+^, Na^+^, K^+^, PO_4_^3−^, SO_4_^2−^, and Cl^−^ on L intensity were observed. FL quenching by SASP was clear but nearly no FL quenching was observed in other ions and biomolecules, indicating that the existence of ions and biomolecules in plasma does not influence the detection of SASP. There was good linearity for SASP concentrations of 0.1–80 µM with an R^2^ of 0.9981 and LOD of 0.04 µM. The developed FL sensor with high accuracy and sensitivity was employed to determine SASP in mouse plasma. The recovery of SASP ranged from 96% to 101% with RSDs from 2.6% to 3.1%. Furthermore, Shahshahanipour and coworkers employed henna leaf CDs as a sensor for the detection of the methotrexate (MTX) drug in plasma [67]. The detection of MTX was performed via the FRET mechanism. For selectivity, the FL response of the sensor to MTX in the presence of co-existing foreign species including Trp, Met, AA, His, glucose, FA, Ca^2+^, Fe^3+^, Mg^2+^, Cu^2+^, K^+^ was examined. The CDs showed significant selectivity for MTX detection. None of the species but MTX were able to establish H-bonding with the CDs to give a proximity condition to the extent necessary for FL quenching, or their adsorption spectrum did not overlap with the emission spectrum of the CDs. In spite of the shared structure of FA and MTX, FA was shown to have a lower FL quenching effect. The FL quenching effects displayed linearity in the range of 0.02 to 18 µM with an R^2^ of 0.987 and LOD of 0.007 µM. The capability of CDs for determining MTX at trace levels in human plasma samples was carried out. The RSD was 1.7% and 2.6% (*n* = 5) for 2.0 and 0.15 µM MTX, respectively. Moreover, Ensafi et al. determined prilocaine (PC) in plasma using saffron-derived CDs [52]. The FL intensity of the CDs was reduced when the PC concentration increased. For selectivity, the sensor response with and without various species such as glucose, Trp, Met, DA, and KCl was examined in the presence of PC. The response of the sensor was highly selective and had an insignificant effect on the sensor response to other species. Linearity was observed with PC concentrations of 0.0023–0.4 µM with an R^2^ of 0.994 and LOD of 0.0018 µM. The RSDs of 1.4% and 2.1% were calculated from 0.0136 and 0.31 µM PC in the blood plasma samples (*n* = 5), respectively.

**Figure 13:**
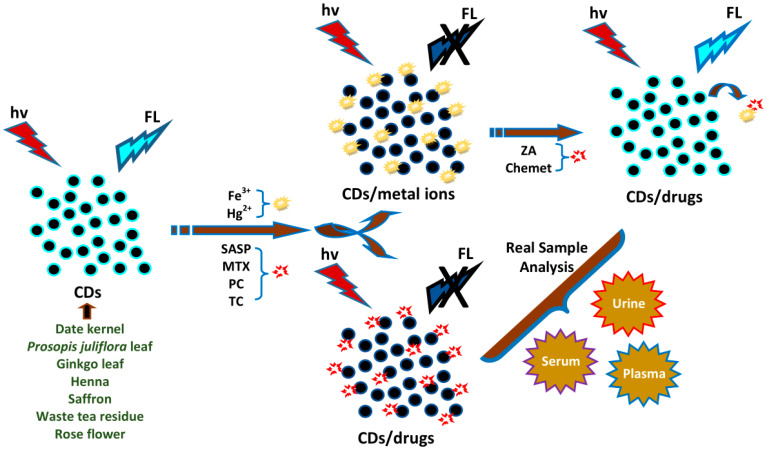
The schematic illustration of plant part-derived CDs for the sensing of drugs in biological samples. The enhancement of FL emission intensity in CD/Fe^3+^ and CD/Hg^2+^ was shown after interactions with ZA and chemet, respectively. The interaction of CDs with SASP, MTX, PC, and TC produced an FL quenching effect. The real sample analysis of all analytes was performed in different biological samples. Abbreviations: ZA, zoledronic acid; SASP, salazosulfapyridine; MTX, methotrexate; PC, prilocaine; TC, tetracycline.

Gunjal et al. used N-CDs derived from waste tea residue for the selective detection of the tetracycline (TC) drug in urine samples [91]. The FL intensity of the CDs decreased progressively with the enhancement of TC concentrations due to the IFE. The selectivity of this FL method was evaluated by using various biomolecules such as GSH, N-acetyl L-cysteine, caffeine, melamine, bovine serum albumin (BSA), Tyr, Cys, DA, etc. No alteration was showen for these biomolecules, indicating the negligible effect of co-existing substances. The addition of TC resulted in a significant FL decrease. The high selectivity of this CD-based FL probe among the other interferons presents a potential ability for the detection of TC in complex samples. Linearity ranged from 0 to 7.2 µM with an LOD of 0.09 µM. The detection of TC in urine samples was employed using the CDs. The TC recovery was 98.41–99.46% with RSDs of 1.45–4.28% in urine samples. Moreover, Feng et al. also determined levels of TC by utilizing CDs from rose flower in human urine samples based on the FL quenching effect [79]. The FL intensity of the CDs reduced to ~50% upon adding 100 µM TC. The quenching of FL intensity was achieved in the presence of TC as well as pH 4.0. The optimum quenching was also seen to be at 50 °C. The selectivity of CDs was explored by examining the response to other substances (aureomycin, oxytetracycline, doxycycline, strepolin, lincomycin, Cys, GSH, His, Vitamin C, glucose, ammonium thiocyanate, ibuprofen, proclaine, and p-aminobenzoic acid). Unlike these substances, TC exhibited high responses to the FL probe, showing the good selectivity of CDs for detecting TC. Linearity ranged from 0.01 to 100 µM TC with an R^2^ of 0.9561 and LOD of 0.0033 µM. The recovery experiments were carried out by using human urine samples. The recoveries of the three samples were 98.3%, 97.8%, and 101.25%, pointing to the potential of using this method for the practical assay of TC in real samples. The schematic illustration of plant part-derived CDs for sensing of all the above drugs in biological samples can be seen in Figure 13.

### 4.9. Sensing of Dyes and Pesticides

Xu and coworkers used aloe CDs for the sensitive detection of tartrazine as an azo-colorant in food samples (steamed buns, honey, and candy) [48]. High FL quenching of the CDs occurred via a static quenching process. Upon adding tartrazine, the FL intensity of the CDs reduced significantly. For the selectivity study, the effect of coexisting foreign substances including Fe^3+^, Ca^2+^, Zn^2+^, K^+^, NO_2_^−^, HCO_3_^−^, GSH, CA, TA, Glu, Phe, starch, sunset yellow, lactose, glucose, Vitamin C, amaranth, and erioglaucine disodium salt was explored on the FL response. Amaranth, sunset yellow, erioglaucine disodium salt, and Fe^3+^ could only be enabled at lower values. However, the concentrations of these substances were lower than the permitted values in food samples. Hence, this strategy showed high selectivity for the detection of tartrazine. The FL quenching efficiency showed linearity with tartrazine concentrations from 0.25 to 32.50 μM with an R^2^ of 0.9986. The RSD was 0.25% at 10 μM tartrazine (*n* = 5). The LOD was estimated to be 0.073 μM. The probe was applied to determine tartrazine at trace level in several food samples. The recoveries were from 88.6% to 103.4% for intraday and from 87.3% to 106.6% for interday testing, respectively. Chatzimitakos et al. also detected tartrazine by using CDs from *Citrus limon* peel [85]. The proposed method was highly selective towards tartrazine compared to other substances including Cd^2+^, Pb^2+^, As^3+^, Ag^+^, Co^2+^, Mn^2+^, Cu^2+^, Sb^3+^, Hg^2+^, Se^2+^, Cr^2+^, Ni^2+^, Fe^3+^, Li^+^, Na^+^, K^+^, Ca^2+^, caffeine, AA, Vitamin B12, FA, Ser, aspartic acid, Leu, Phe, Met, Ala, Gly, Ile, Glu, and Tyr, which did not cause significant quenching (<5%). The method was selective for tartrazine since 25 μM of the tartrazine induced approximately 60% FL quenching. The FL emission intensity decreased upon the addition of tartrazine of up to 80 μM. The quenching induced by tartrazine showed a linear response between 0.6 and 23.5 μΜ (R^2^ = 0.9936, LOD = 0.2 μM). The RSD was 2.3% of 23 μM tartrazine for intraday and 3.4% for interday measurements (*n* = 3). The application of this probe was verified by examining food products including energy drinks, juice, and ice cream. Good recoveries were reached between 95.0% and 99.9%, confirming the applicability of this method to real samples.

**Figure 14:**
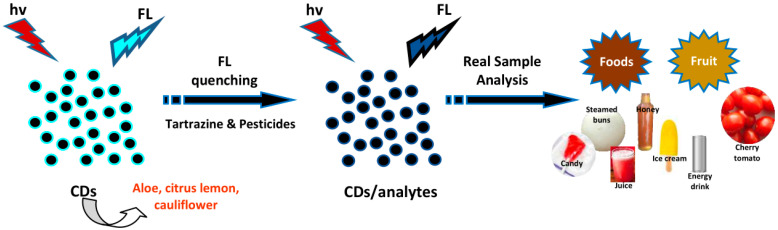
Schematic illustration of plant part-derived CDs for the sensing of tartrazine dye and pesticides. The FL emission intensities of CDs were quenched after adding tartrazine and pesticides. The detection of these analytes was utilized in foods and fruit samples.

Tafreshi et al. performed the ultrasensitive FL determination of pesticides including diazinon, glyphosate, and amicarbazone in cherry tomatoes using CDs from cauliflower juice [53]. The combination of CDs and pesticides produced FL emission quenching. A significant quenching in the FL intensity of CDs could be viewed with the naked eye under UV light. The specificity was evaluated by observing the alteration of FL emission intensity of the CDs in the presence of bromacil and dialen super pesticides. These pesticides did not notably affect the CDs’ FL intensity. By enhancing the concentration, the reducing intensity exhibited insignificant alteration compared to diazinon, amicarbazone, and glyphosate. The FL intensity of CD/diazinon was significantly quenched with an increase of diazinon concentration from 0.00082 to 16.43 µM. The quenching efficiency of glyphosate was lower than that of diazinon. The emission peaks of the CDs quenched significantly with increasing glyphosate concentration ranging from 0.012 to 29.57 µM. Significant quenching was observed in the FL intensity with increases in amicarbazone concentration (0.0021–20.72 µM). Then, the FL sensing characteristics of CDs for diazinon, glyphosate, and amicarbazone in free cherry tomatoes were tested. Diazinon, glyphosate, and amicarbazone exhibited a quenching effect on the existence of CDs in tomato juices. By enhancing the concentration of pesticides in CDs from cherry tomato juices, the FL emission intensity was significantly quenched. The LODs were calculated to be 0.00082, 0.0021, and 0.012 µM for diazinon, amicarbazone, and glyphosate, respectively. The schematic illustration of plant part-derived CDs for the sensing of dye and pesticides is depicted in Figure 14.

### 4.10. Sensing of Nitrite and Borax

**Figure 15:**
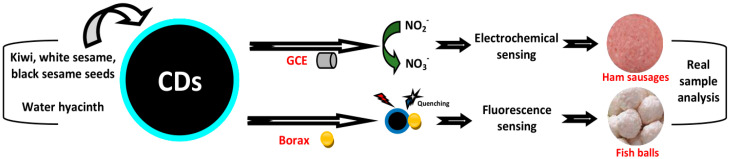
Schematic illustration of plant part-derived CDs for the sensing of nitrite and borax. The detection of nitrite was performed via electrochemical sensing while the detection of borax was performed via FL sensing. The detection of these analytes was also assessed in food samples.

Li et al. used CDs from kiwi, white sesame, and black sesame seeds for the electrochemical sensing of NO_2_^−^ (nitrite) in ham sausages [84]. A bare glass carbon electrode (GCE) and a CD/GCE combination exhibited nearly no electrochemical sensitivity without the addition of nitrite at a scan rate of 20 mV·s^−1^. After the addition of 2 mM nitrite, the current responses were progressively enhanced at numerous modified electrodes and the bare GCE. All catalytic current peaks on the CD/GCE were increased and the overpotential for nitrite oxidation was lowered. The response current and potential were enhanced compared to that on a bare GCE, demonstrating that the existence of CDs showed good catalytic work for nitrite oxidation. The electrocatalytic influence of Nafion film on nitrite was very low, indicating that Nafion is a desired film for electrode modification. When nitrite was inserted into a 0.1 M acetate buffer solution at pH 5.0, an oxidation peak could be viewed. The oxidation current progressively increased with the increase of nitrite concentration, showing that CDs have good catalytic work towards nitrite. All of the anodic peak potentials of nitrite at CD/GCEs were about +0.79 V vs. Ag/AgCl. To investigate the selectivity of nitrite detection, several substances including AA, lactose, glucose, UA, Na_2_HPO_4_, and KCl were introduced and determined by testing the differential pulse voltammetry (DPV) responses on CD/GCE in acetate buffer with 0.2 mM nitrite. These possibly coexisting chemicals had a negligible effect on the detection of nitrite when utilized as an electrode modification material. Upon the addition of nitrite, the peak current of DPV enhanced clearly as the concentration of nitrite increased, as did the sensitive current response at lower nitrite concentrations. The N-CD1-and N-CD2-modified electrodes showed a broad linearity in the nitrite concentration ranges of 0.7–6000 μM and 0.7–8000 μM with an R^2^ of 0.9994 and 0.9980, respectively. Meanwhile, for the N-CD3-modified electrode, the linearity range was generally narrow from 0.7 to 2000 μM with an R^2^ of 0.9975. The LOD of the nitrite detection was 0.23 μM, demonstrating the good catalytic performance of N-CDs. The recoveries of nitrite were 95.8–104.0%, 97.3–103.2%, and 96.4–105.0% using N-CD1, N-CD2, and N-CD3, respectively, demonstrating that the sensors were appropriate for nitrite detection in ham sausages. Finally, Prathumsuwan [86] used CDs prepared from water hyacinth for the sensing of borax in real fish ball samples. Their application to detect borax was demonstrated with a LOD of 1.5 μM. The selective and sensitive response of CDs for borax in the presence of disturbances assured their specific detection. The selective determination of borax by CDs was explored by using several species such as Ca^2+^, K^+^, Na^+^, Li^+^, PO_4_^3−^, CO_3_^2−^, SO_4_^2−^, I^−^, Cl^−^, ClO_4_^−^, CH_3_COO^−^, NO_3_^−^, AA, CA, monosodium glutamate, glucose, and boric acid, introduced into a CD–borax solution. None of the analytes had significant effect on the FL emission of the CD–borax solution except AA and CA. The CDs showed selectivity to borax even in the presence of interference. The small FL quenching by AA and CA was due to the increased acidity of the solutions. The pH-dependent FL emission of both the CDs and the CD–borax solution indicated lower emission in the acidic range. A portable paper-based device was prepared for utilization in on-site borax determination with an LOD of 11.85 μM. Real fish ball samples were examined and showed good borax recovery in concentrations ranging from 98.8% to 101.8%. A schematic illustration of plant part-derived CDs for the sensing of nitrite and borax in real samples can be seen in Figure 15. The analytical application performance of plant part-derived CDs for the sensing of metal ions, drugs, colorant, pesticides, nitrite, and borax is summarized in Table 5.

## 5. Summary and Perspectives

Herein, the different synthesis methods, optical properties, and recent applications of CDs as biosensors were discussed. CDs synthesized from various plant parts including stems, flowers, fruits, leaves, seeds, and roots with the synthesis methods of hydrothermal, solvothermal, microwave, and microwave-assisted hydrothermal methods, pyrolysis, and chemical oxidation were overviewed. Most plant part-derived CDs eliminate the need for surface-chemical passivating agents, as they contain various functional groups. The heteroatom doping obtained by self-passivation provides an excellent effect on their optical properties, generating powerful applications. The optical properties of plant part-derived CDs including FL and UV-vis absorbance were enumerated. The CDs have proven their benefits in biosensing applications due to their easy synthesis, low cytotoxicity, and superior optical properties. The biosensing applications of plant part-derived CDs in the sensing of bioactive molecules (Cys, AA, GSH, D-PA, thiamine, riboflavin, CoA, HA/HAase, *E. coli*, hemin, DNA, ATP, CEA, and CEA-aptamer) and molecules (Pb^2+^, Hg^2+^, V^5+^, Fe^3+^, tartrazine, ZA, chemet, SASP, MTX, PC, TC, diazinon, glyphosate, amicarbazone, nitrite, and borax) in biological samples have been systematically stated according to their target categories. The selectivity and sensitivity of CDs will broaden and enrich their biosensing applications. Plant part-derived CDs show excellent sensitivity and selectivity to various target analytes, especially for biomolecules. The apparent advances in the improvement of plant part-derived CDs for biosensing truly mark it out as a future material and distinguish it from other carbon materials. Most of the biosensing applications of plant part-derived CDs are based on FL sensors. Despite plentiful opportunities in the field of plant part-derived CDs, there are still many challenges in exploring the tremendous potency of plant part-derived CDs in future:

1. CDs should be synthesized from other low-cost waste plant parts or other natural sources.
2. In addition to the hydrothermal method, other synthesis methods could be useful for synthesizing plant part-derived CDs.
3. The utilization of solid waste residues after separating CDs is needed to explore more beneficial materials.
4. New natural resources containing N, S, P, or other heteroatom elements should be explored more in the future.
5. An effective synthesis method with a high QY should be developed to fabricate CDs.
6. The exploration of plant part-derived CDs for other biomolecules should be investigated.
7. The large precursor compositions might lead to heterogeneity in plant part-derived CDs and a focused investigation of separation and purification is required.
8. There is no literature on the in vivo application of plant part-derived CDs to detect biologically important small molecules in exposed living things. Therefore, increased attention is required in this field.

## Figures and Tables

**Table 1:** The features of several methods for the synthesis of plant part-derived CDs.

Methods	Advantages	Disadvantages
Hydrothermal/Solvothermal	Low cost, eco-friendly, non-toxic, simple	Poor control of size, long synthesis duration
Microwave/Microwave-Hydrothermal	Facile, rapid, scalable, low cost, eco-friendly, treamlined process	Poor control of size
Pyrolysis	Simple, short synthesis duration, ecofriendly	Difficult to scale up, broad size distribution
Chemical Oxidation	Cheap, large-scale production, effective	Tedious steps, toxic acid/base reagents, expensive oxidants, complicated post-treatment

**Table 2:** The synthesis conditions of plant part-derived CDs through the hydrothermal method.

Precursor	Part	Form	Amount	Solvent (mL)	Temp (°C)	Time (h)	Ref.
Aloe	Stem	Powder	5.0 g	25	180	11	[48]
*Sargassum fluitans*	Stem	Extract	10 mL	50	180	5	[49]
Mushroom	Stem	Powder	0.6 g	6	200	6	[50]
Shiitake mushroom	Stem	Powder	0.5 g	10	200	12	[51]
Saffron	Flower	Powder	0.5 g	100	200	14	[52]
Cauliflower juice	Flower	Extract	60 mL	-	120	5	[53]
Groundnut	Seed	Powder	1.0 g	30	250	6	[54]
Mustard	Seed	Powder	2.0 g	50	180	4	[55]
Water chestnut/Onion	Root	Powder	2.0/3.0 g	30	180	4	[56]
Tomato juice	Fruit	Extract	10 mL	-	150	2	[57]
Papaya flesh	Fruit	Powder	0.4 g	10	200	5	[58]
Lemon juice	Fruit	Extract	15 mL	10	240	12	[59]
Lemon/grape/turmeric + EDA	Fruit	Extract	1.0 g	40	180	6	[60]
*Lantana camara* berry + EDA	Fruit	Extract	5.0 g	50	180	3	[61]
*Lycii fructus* + NH_3_	Fruit	Powder	1.0 g	30	200	5	[62]
Date kernel	Fruit	Powder	2.0 g	10	200	8	[63]
Pineapple peel juice	Fruit	Extract	10 mL	10	150	2	[64]
Watermelon juice	Fruit	Extract	50 mL	5	180	3	[65]
Ginkgo leaf	Leaf	Powder	1.0 g	25	200	10	[66]
Henna	Leaf	Powder	0.5 g	40	180	12	[67]
Scallion	Leaf	Powder	4.0 g	20	180	12	[68]
*Azadirachta indica* (neem)	Leaf	Powder	10 g	100	150	4	[69]
Waste tea + EDA	Leaf	Extract	1.5 g	30	150	6	[70]
Mint	Leaf	Extract	5.0 g	40	200	5	[71]

**Table 3:** The optical properties of plant part-derived CDs as biosensors. Abbreviations: λ_Ex_, excitation wavelength; λ_Em_, emission wavelength; QY, quantum yield, Abs, absorbance; B/G/Y, blue/green/yellow.

Precursors	λ_Ex_ (nm)	λ_Em_ (nm)	QY (%)	Abs. (nm)	FL Color	Ref.
Aloe	441	503	10.37	278	Bright blue	[48]
*Sargassum fluitans*	340	450	18.2	226/280	Blue	[49]
Mushroom	370	455	15.3	285	Blue	[50]
Shiitake mushroom	330	410	5.5	240–290/420–500	Bright blue	[51]
Saffron	400	485	23.6	275	Green-blue	[52]
Cauliflower	325	400	43	280	Blue	[53]
*Bauhinia* flower	355	442	27	228/282	Blue	[77]
Rose flower	390	435	13.45	-	Blue	[79]
Mahogany fruit shell	320	430	1.9	300	-	[90]
Coconut water	390	450	2.8	290	Blue/green	[81]
Tomato juice	367	440	13.9	-	Blue	[57]
Tomato	360/420/460	450/520/560	12.7	260/280/285	B/G/Y	[93]
Papaya flesh	370	450	18.98	250–290	Blue	[58]
Lemon/grape/turmeric/EDA	-	-	20	350	Blue	[60]
*L. camara* berry/EDA	360	450	33.15	285/356	Blue	[61]
Muskmelon	342/415/425	432/515/554	14.3	314/414/467	B/G/Y	[92]
Lemon juice	420	540	21	280	Bright green	[59]
*Lycii fructus* + NH_3_	350	430	17.2	271/300	Blue	[62]
Date kernel	340	430	12.5	275	Blue	[63]
Pineapple Peel	380	435	42	280	Blue	[64]
Pineapple	318/395/393	438/516/543	44.7	318/395/393	B/G/Y	[94]
Watermelon juice	355	439	10.6	282/355	Blue	[65]
*Citrus sinensis/limon* peels	365/330	455/390, 435	16.8/15.5	270	Blue	[85]
Lemon/Onion/NH_3_	340	425	23.6	280/340	Bright blue	[78]
Ginkgo leaf	350	436	22.8	230/280	Bright blue	[66]
Waste tea residue	310	430	2.47	302	-	[91]
Henna	360	440	28.7	270–380	Green	[67]
Scallion	320	418	3.2	281	Blue	[68]
*Azadirachta indica* (neem)	340	467	27.2	276/340	Blue	[69]
*Prosopis juliflora*	325/350	396/437	5	300–500	Bright blue	[83]
Waste tea/EDA	350	445	7.1	270/330	Blue	[70]
Mint	360	441	7.64	225/281/323	Cyan	[71]
Water hyacinth	400	486	27	285/350	Blue	[86]
White pepper	420	520/668	10.4	261/310/343/665	-	[74]
Groundnut	360	443	7.87	279	-	[54]
Kiwi, white & black sesame	-	-	-	275/325	-	[84]
Mustard	330	423	4.6	245/312	Blue	[55]
Water Chestnut/Onion	370	475	12	242/333	Green-blue	[56]

**Table 4:** The applications of plant part-derived CDs for the sensing of various biomolecules. Abbreviations: LOD, limit of detection; F+R, fruit+root; cfu, colony-forming unit.

Sources	Parts	Target Analytes	LOD (µM)	Ref.
Pineapple Peel	Fruit	Cys	0.87	[64]
Watermelon juice	Fruit	Cys	0.27	[65]
Waste tea + EDA	Leaf	Cys & AA	8.785/153.5 & 87.02/19.78	[70]
Groundnut	Seed	GSH	-	[54]
Mahogany fruit shell	Fruit	D-PA	49.59/39.27	[90]
*Azadirachta indica*	Leaf	AA	1.773	[69]
Mustard	Seed	AA	3.26	[55]
Mint	Leaf	AA	0.079	[71]
Coconut water	Fruit	Thiamine	0.28	[81]
Lemon/Onion/NH_3_	F + R	Riboflavin	0.003	[78]
White pepper	Seed	CoA	0.00875	[74]
Water Chestnut/Onion	Root	CoA	0.01	[56]
Mushroom	Stem	HA/HAase	0.3 × 10^−5^/0.1 U mL^−1^	[50]
Papaya flesh	Fruit	*E. coli*	9.5 × 10^4^ cfu mL^−1^	[58]
Lemon/grape/turmeric+EDA	Fruit	*E. coli*	-	[60]
Scallion	Leaf	Hemin	0.1	[68]
Shiitake mushroom	Stem	Hemin	0.12	[51]
*Sargassum fluitans*	Stem	DNA	15.15	[49]
*Bauhinia* flower	Flower	ATP	0.005	[77]
Tomato juice	Fruit	CEA & aptamer	0.3 ng mL^−1^/0.00188	[57]

**Table 5:** The applications of plant part-derived CDs for the sensing of various molecules in biological samples.

Sources	Parts	Analytes	LOD (µM)	Biological Samples	Ref.
*L. camara* berry + EDA	Fruit	Pb^2+^	0.00964	Serum/Urine	[61]
Muskmelon	Fruit	Hg^2+^	0.33	Serum	[92]
Lemon juice	Fruit	V^5+^	27.36	Fetal Bovine Serum	[59]
*Lycii fructus* + NH_3_	Fruit	Fe^3+^	0.021	Urine	[62]
Tomato	Fruit	Fe^3+^	0.016, 0.072, 0.065	Plasma/urine	[93]
Pineapple	Fruit	Fe^3+^	0.03	Plasma/urine	[94]
*Citrus sinensis/limon* peels	Fruit	Fe^3+^/Tartrazine	0.003/0.2	Blood/urine	[85]
Date kernel	Fruit	ZA	0.04	Serum	[63]
*Prosopis juliflora*	Leaf	Chemet	0.0077	Serum	[83]
Ginkgo leaf	Leaf	SASP	0.04	Mouse plasma	[66]
Henna	Leaf	MTX	0.007	Plasma	[67]
Saffron	Flower	PC	0.0018	Plasma	[52]
Waste tea residue	Leaf	TC	0.09	Urine	[91]
Rose flower	Flower	TC	0.0033	Urine	[79]
Aloe	Stem	Tartrazine	0.073	Foods	[48]
Cauliflower	Flower	Pesticides	0.00082, 0.0021, 0.012	Cherry tomato	[53]
Kiwi/white & black sesame	Seed	NO_2_^−^	0.23	Ham sausage	[84]
Water hyacinth	Leaf	Borax	1.5/11.85	Fishball	[86]
